# The structure of Prp2 bound to RNA and ADP-BeF_3_^−^ reveals structural features important for RNA unwinding by DEAH-box ATPases

**DOI:** 10.1107/S2059798321001194

**Published:** 2021-03-30

**Authors:** Florian Hamann, Lars C. Zimmerningkat, Robert A. Becker, Tim B. Garbers, Piotr Neumann, Jochen S. Hub, Ralf Ficner

**Affiliations:** a Department of Molecular Structural Biology, Institute of Microbiology and Genetics, Göttingen Center for Molecular Biosciences (GZMB), Georg-August-University Göttingen, Justus-von-Liebig-Weg 11, 37077 Göttingen, Germany; b Cluster of Excellence ‘Multiscale Bioimaging: from Molecular Machines to Networks of Excitable Cells’ (MBExC), Georg-August-University Göttingen, Göttingen, Germany; c Theoretical Physics and Center for Biophysics, Saarland University, Saarbrücken, Germany

**Keywords:** spliceosome, RNA helicases, DEAH-box ATPases, Prp2, Prp43

## Abstract

Noncoding intron sequences present in precursor mRNAs need to be removed prior to translation, and they are excised *via* the spliceosome, a multimegadalton molecular machine composed of numerous protein and RNA components. The DEAH-box ATPase Prp2 plays a crucial role during pre-mRNA splicing as it ensures the catalytic activation of the spliceosome. Despite high structural similarity to other spliceosomal DEAH-box helicases, Prp2 does not seem to function as an RNA helicase, but rather as an RNA-dependent ribonucleo­protein particle-modifying ATPase. Recent crystal structures of the spliceosomal DEAH-box ATPases Prp43 and Prp22, as well as of the related RNA helicase MLE, in complex with RNA have contributed to a better understanding of how RNA binding and processivity might be achieved in this helicase family. In order to shed light onto the divergent manner of function of Prp2, an N-terminally truncated construct of *Chaetomium thermophilum* Prp2 was crystallized in the presence of ADP-BeF_3_^−^ and a poly-U_12_ RNA. The refined structure revealed a virtually identical conformation of the helicase core compared with the ADP-BeF_3_^−^- and RNA-bound structure of Prp43, and only a minor shift of the C-terminal domains. However, Prp2 and Prp43 differ in the hook-loop and a loop of the helix-bundle domain, which interacts with the hook-loop and evokes a different RNA conformation immediately after the 3′ stack. On replacing these loop residues in Prp43 by the Prp2 sequence, the unwinding activity of Prp43 was abolished. Furthermore, a putative exit tunnel for the γ-phosphate after ATP hydrolysis could be identified in one of the Prp2 structures.

## Abstract 2

The crystal structure of *Chaetomium thermophilum* Prp2 bound to ADP-BeF_3_^−^ and a poly-U_12_ RNA reveals a conserved pre-catalytic helicase conformation and only minor shifts of the C-terminal domains. Differences in the hook-loop and a loop of the helix-bundle domain compared with Prp43 evoke divergent conformations of the bound RNA, ultimately impacting the ability to unwind double-stranded RNA.

## 1. Introduction   

In eukaryotes, most precursor messenger RNAs (pre-mRNAs) contain noncoding intron sequences which need to be removed in order to obtain a mature mRNA that can serve as a template for translation. The vast majority of these intervening sequences are removed with the help of the spliceosome (Will & Lührmann, 2011 ▸; Wahl *et al.*, 2009 ▸; Matera & Wang, 2014 ▸). The spliceosome is a multimegadalton molecular machine that is sequentially assembled from RNA and protein components. For each intron to be removed, the complex is formed *de novo* on a pre-mRNA. Since it has no preformed active site, compositional as well as conformational rearrangements ensure the formation of a catalytically active complex. Once the intron has been excised via two subsequent transesterification reactions, the complex is completely dis­assembled and each component is available for a new round of splicing.

These rearrangements need to be tightly orchestrated and are driven by a set of so-called DE*x*D/H-box helicases that ensure the transition of the various spliceosomal complexes (Cordin *et al.*, 2012 ▸; Ding & Pyle, 2012 ▸; Ozgur *et al.*, 2015 ▸). The assembly steps are dominated by DEAD-box helicases, followed by the Ski2-like helicase Brr2, and all subsequent activation, catalytic and disassembly steps are performed by DEAH-box ATPases. All of them belong to helicase superfamily 2 (SF2) and have a helicase core composed of two RecA-like domains which harbor at least eight conserved sequence motifs (I, Ia, Ib, II, III, IV, V and VI; Fairman-Williams *et al.*, 2010 ▸). They play important roles in ATP binding and hydrolysis, in RNA binding and in coupling these processes to unwinding/translocation (Schwer & Meszaros, 2000 ▸; Campodonico & Schwer, 2002 ▸; Schneider *et al.*, 2004 ▸). Additionally, Ski2-like and DEAH-box ATPases possess an auxiliary C-terminal domain that forms an RNA-binding tunnel together with the helicase core (Büttner *et al.*, 2007 ▸; He *et al.*, 2017 ▸; Prabu *et al.*, 2015 ▸; Tauchert *et al.*, 2017 ▸; Hamann *et al.*, 2019 ▸).

One key player during the catalytic activation of the spliceosome is the DEAH-box ATPase Prp2, which ensures the transition from the B^act^ to the B* complex (King & Beggs, 1990 ▸; Roy *et al.*, 1995 ▸; Kim & Lin, 1996 ▸; Silverman *et al.*, 2004 ▸). Here, it is responsible for destabilization of the SF3a/b complex, which exposes the 5′ splice site and the branch site, enabling the first transesterification step (Ohrt *et al.*, 2012 ▸; Lardelli *et al.*, 2010 ▸; Warkocki *et al.*, 2009 ▸; Bao *et al.*, 2017 ▸). For its function, it is strictly required to interact with the G-patch protein Spp2 (Roy *et al.*, 1995 ▸; Silverman *et al.*, 2004 ▸; Warkocki *et al.*, 2015 ▸; Krishnan *et al.*, 2013 ▸; Hamann *et al.*, 2020 ▸). Interestingly, Prp2 is the only spliceosomal DEAH-box ATPase that does not show any RNA-unwinding activity *in vitro* (Tauchert *et al.*, 2017 ▸; Tanaka *et al.*, 2007 ▸; Christian *et al.*, 2014 ▸; Schwer & Gross, 1998 ▸; Tanaka & Schwer, 2005 ▸; Wang *et al.*, 1998 ▸). While recent biochemical evidence and cryo-EM structures suggest that DEAH-box ATPases are likely to act as translocases rather than unwindases in the spliceosome, Prp16, Prp22 and Prp43 still have the ability to unwind double-stranded RNA (Liu *et al.*, 2017 ▸; Rauhut *et al.*, 2016 ▸; Galej *et al.*, 2016 ▸; Yan *et al.*, 2016 ▸).

In order to investigate why Prp2 is not able to unwind RNA duplexes like other closely related spliceosomal DEAH-box ATPases, we crystallized Prp2 in the presence of RNA and the ATP analog ADP-BeF_3_^−^. Comparing this structure with ADP-bound Prp2 structures, we were able to identify an as yet undescribed tunnel between the nucleotide-binding site and the protein surface which could serve as an exit passage for the hydrolyzed γ-phosphate. The opening of this channel is mediated by movement of the conserved sequence motif III. Additionally, we could observe an alternative mode of binding of the RNA in Prp2 with a kink in the RNA backbone, which is shifted in position compared with RNA-bound Prp22 and Prp43 structures. We identified a loop in the C-terminal domains to play an important role in threading the 5′ RNA region, which differs strongly between Prp2 and Prp43/Prp22. We postulate that this difference in RNA binding due to the influence of the C-terminal loop impedes Prp2 from being a competent unwindase.

## 2. Materials and methods   

### 2.1. Protein production and crystallization   

Prp2 from *Chaetomium thermophilum* (ctPrp2) containing residues 286–921 was recombinantly produced in *Escherichia coli* Rosetta2 (DE3) cells as a GST-tag fusion protein at 16°C using an autoinduction protocol (Studier, 2014 ▸) and purified as described in Hamann *et al.* (2020 ▸). ctPrp2 was stored and used for crystallization in the following buffer: 10 m*M* Tris–HCl pH 7.5, 200 m*M* NaCl, 5%(*v*/*v*) glycerol, 2 m*M* MgCl_2_. ctPrp43(61–764) mutants and ctPfa1(662–742) (ctPfa1-GP) were recombinantly expressed and purified as specified in Tauchert *et al.* (2016 ▸, 2017 ▸).

A solution consisting of 2 mg ml^−1^ ctPrp2 (27.4 µ*M*), a tenfold molar excess of ADP (274 µ*M*), a 20-fold molar excess of BeSO_4_ (548 µ*M*), a 60-fold molar excess of NaF (1.644 m*M*) and a 2.5-fold molar excess of U_12_ ssRNA (68.5 µ*M*; Axolabs, Germany) was incubated for at least 30 min at 4°C prior to crystallization trials. The complex was crystallized using the sitting-drop vapor-diffusion technique by mixing 1 µl complex solution with 1 µl crystallization buffer. Crystals were grown in 100 m*M* MOPS/Na HEPES pH 7.5, 8% PEG 20 000, 22% PEG MME 550, 20 m*M* 1,6-hexanediol, 1-butanol, (*RS*)-1,2-propanediol, 2-propanol, 1,4-butanediol and 1,3-propanediol. Rod-shaped crystals were obtained after two days of incubation at 20°C.

### 2.2. Data collection and processing   

Prior to data collection, the crystals were cryoprotected with reservoir solution complemented with 5%(*v*/*v*) PEG 400 and 5%(*v*/*v*) glycerol and flash-cooled in liquid nitrogen for storage. Oscillation images were collected at 100 K on beamline P14 at PETRA III, DESY, Hamburg, Germany using an oscillation range of 0.1° and an exposure time of 0.01 s per image. Data processing was performed using the *XDS* package (Kabsch, 2010 ▸). The data did not appear to be twinned, and no significant pseudo-translation could be detected as reported by *phenix.xtriage*. The results of the *L*-test indicate that the intensity statistics behave as expected and no twinning is suspected. X-ray diffraction data statistics are summarized in Table 1 ▸.

### 2.3. Structure solution, refinement and analysis   

The structure of ctPrp2 in complex with ADP-BeF_3_^−^ and poly-U RNA was solved by molecular replacement using *Phaser* (McCoy *et al.*, 2007 ▸). The RecA1, RecA2 and C-terminal domains of the ADP-bound ctPrp2 structure (PDB entry 6fa5; Schmitt *et al.*, 2018 ▸) were used as individual search models for molecular replacement. Due to the divergent conformation of Prp2 in this complex, no phasing solution could be found using an existing complete model. The model was manually built with *Coot* (Emsley *et al.*, 2011 ▸) and refinement was performed with *Phenix* (Liebschner *et al.*, 2019 ▸), including TLS, weight optimization and bulk-solvent optimization. The validation tools in *Phenix* and *MolProbity* were used to assess the final model quality (Chen *et al.*, 2010 ▸). A maximum-likelihood-based coordinate error of 0.20 for the final model was estimated by *Phenix*. Superpositions of structures were performed with *LSQMAN* (Kleywegt, 1996 ▸) and figures were prepared with *PyMOL* (version 1.8, Schrödinger).

### 2.4. Molecular dynamics   

Molecular-dynamics (MD) simulations of the release of phosphate from the ATP-binding pocket were set up as follows. In the simulations, the presented Prp2 structure and the structure of Prp43 (PDB entry 5lta; Tauchert *et al.*, 2017 ▸) were used, both representing the complex of the respective enzyme with U_7_ RNA and the ATP analog ADP-BeF_3_^−^. The ATP analog was replaced with ADP and dihydrogen phosphate (DHP). The structure was placed into a simulation box with the shape of a dodecahedron. The box was solvated with 24 798 water molecules for Prp2 and 36 616 water molecules for Prp43. Each system was then neutralized by 13 potassium counter-ions. Interactions of protein and RNA were described with the Amber14SB force field (Maier *et al.*, 2015 ▸). Water was modeled with the TIP3P model (Jorgensen *et al.*, 1983 ▸). Parameters for ADP and DHP were taken from Meagher *et al.* (2003 ▸) and Kashefolgheta & Vila Verde (2017 ▸), respectively, and were translated into GROMACS format with the *ACPYPE* software (Sousa da Silva & Vranken, 2012 ▸). The parameters for K^+^ were taken from Joung & Cheatham (2008 ▸). For the interactions between Mg^2+^ and DHP:O (the negative-charged O atom of DHP), the combination rule for Lennard–Jones interactions was overwritten with the nonbonded interactions suggested by Panteva *et al.* (2015 ▸). The energy of the system was minimized with the steepest-descent algorithm. The system was then equilibrated for 100 ps with positional restraints acting on the heavy atoms, including RNA, ADP and DHP (*k* = 1000 kJ mol^−1^ nm^−2^).

Electrostatic interactions were described with the particle mesh Ewald. Dispersion interactions and short-range repulsion were described together using a Lennard–Jones potential with a cutoff at 1 nm. The temperature was controlled at 300 K using velocity scaling (Bussi *et al.*, 2007 ▸) by coupling protein/RNA/ADP/DHP and water/K^+^ to two separate heat baths (τ = 0.5 ps). The pressure was controlled at 1 bar with the Parrinello–Rahman barostat (τ = 5 ps; Parrinello & Rahman, 1981 ▸). An integration time step of 2 fs was used. The geometry of water molecules was constrained with *SETTLE* (Miyamoto & Kollman, 1992 ▸), while all other bonds were constrained with *P-LINCS* (Hess, 2008 ▸).

To accelerate the dissociation of DHP from the complex, we used random-accelerated MD simulations (RAMD; Lüdemann *et al.*, 2000 ▸). The *GROMACS* code (version 2020.1) extended for RAMD was taken from https://github.com/HITS-MCM/gromacs-ramd (Kokh *et al.*, 2020 ▸). For the simulations reported in this study, we used the following RAMD settings. Mg^2+^ and DPH were considered as the receptor and the ligand, respectively. An accelerating force of 585.2 kJ mol^−1^ nm^−1^ was used, and simulations were evaluated every 50 steps. A different random seed was used for each simulation. If the ligand had traveled less than 0.005 nm within 50 steps, the direction of force was changed. The simulation stopped at a ligand–receptor distance of 4 nm. For Prp2 30 RAMD simulations were performed with these parameters, and 15 RAMD simulations were carried out for Prp43. In addition, we tested 5 RAMD simulations with a force of 635 kJ mol^−1^ nm^−1^ and 5 RAMD simulations with a force of 700 kJ mol^−1^ nm^−1^ for Prp43. Notably, we tested various alternative RAMD settings; in the case of successful dissociation events, these simulations revealed similar DHP-exit pathways.

### 2.5. ATPase activity assay   

The ATPase activities of various ctPrp43 mutants were tested using a nicotinamide adenine dinucleotide (NADH)-dependent coupled enzymatic assay (Agarwal *et al.*, 1978 ▸). ATP consumption has a direct effect on the decrease in the NADH absorption at 340 nm, which was recorded over time with a VICTOR Nivo Multimode Microplate Reader (Perkin Elmer). All reactions were performed in triplicates of 150 µl each at 25°C in 25 m*M* Tris–HCl pH 7.5, 150 m*M* KCl, 3 m*M* MgCl_2_ supplemented with 250 µ*M* NADH, 500 n*M* phosphoenolpyruvate, 6–8.3 U ml^−1^ pyruvate kinase, 9–14 U ml^−1^ lactate dehydrogenase and 2 m*M* ATP. Measurements in the presence of A_20_-ssRNA (Axolabs, Germany) or/and ctPfa1-GP were conducted with a tenfold and a fivefold molar excess, respectively. All ctPrp43 mutants were used at a concentration of 0.5 µ*M*. The ATP consumption per minute (*k*_obs_) was calculated using
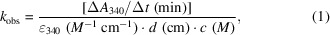
where Δ*A*_430_/Δ*t* is the slope of the NADH decrease, ɛ_340_ is the extinction coefficient of NADH, *d* is the optical pathlength and *c* is the protein concentration.

### 2.6. Helicase activity assay   

A fluorescence-based unwinding assay was used to monitor the helicase activities of various ctPrp43 mutants (Tauchert *et al.*, 2017 ▸; Christian *et al.*, 2014 ▸; Belon & Frick, 2008 ▸). The disruption of a dsRNA substrate with a 3′-ssRNA overhang, consisting of 5′-GCG CCU ACG GAG CUG GUG GCG UAG GCG CAA AAA AAA AAA AAA AAA AAA-3′ and 5′-(Cy5)-GCG CCU ACG CCA CCA GCU CCG UAG GCG C-(BBQ)-3′, was measured by tracking the decrease in fluorescence due to the quenching of Cy5 by BBQ. Upon unwinding, the labeled RNA strand forms an internal hairpin, which brings BBQ and Cy5 into close proximity, leading to the quenching. Measurements were performed in 25 m*M* Tris–HCl pH 7.5, 150 m*M* KCl, 3 m*M* MgCl_2_, 1 m*M* ATP at 25°C and were recorded with a VICTOR Nivo Multimode Microplate Reader (Perkin Elmer). ctPrp43 mutants were used at a concentration of 0.25 n*M*, ctPfa1-GP at 1.25 µ*M* and the 3′-overhang dsRNA (Axolabs, Germany) at 500 n*M*. The excitation wavelength was set to 640 nm and the emission was measured at 685 nm. The helicase reaction speed was calculated by determining the initial slope (20–110 s) of each reaction, which represents the maximum reaction velocity (Supplementary Fig. S1*a*). The intersection of the initial reaction slope with the fluorescence signal of the unwound hairpin RNA was used to determine the time it would take to unwind 500 nmol of dsRNA at this initial maximum reaction velocity. The rate of unwinding of one dsRNA (*k*_obs_) was calculated by subsequently accounting for the protein concentration used for each measurement. A detailed description of one example of the calculation of the reaction rate constants is provided in Supplementary Fig. S1(*b*). Three independent measurements were conducted per sample and the reaction rate constants of the ctPrp43 mutants are plotted with the corresponding standard deviations.

## 3. Results   

### 3.1. Overall conformation of ADP-BeF_3_^−^- and RNA-bound Prp2   

**Figure 1:**
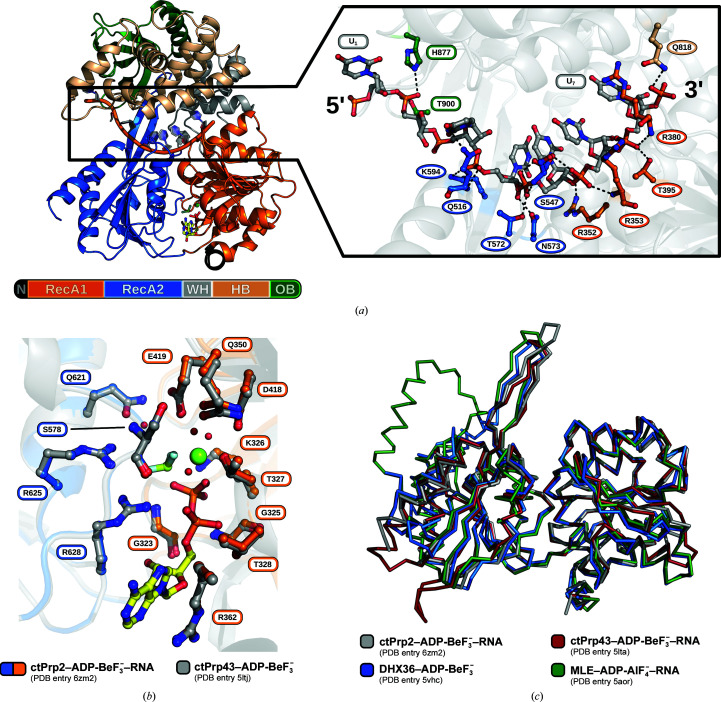
Structural overview of the pre-catalytic state of Prp2. (*a*) Prp2 and the bound RNA are displayed as a cartoon model and ADP-BeF_3_^−^ is depicted as sticks. The crystallized construct is composed of two RecA-like domains (RecA1, orange; RecA2, blue), a winged-helix (WH) domain (gray), a helix-bundle (HB) domain (wheat) and a oligosaccharide-binding (OB) domain (green). The RNA is bound between the helicase core and the C-terminal domains and the nucleotide is sandwiched between the RecA-like domains. Conserved residues interacting with the RNA backbone of the 3′ stacked region are shown in circles, whereas the remaining interacting residues are shown in rectangular shapes. (*b*) Superposition of active-site residues of Prp2 and Prp43 interacting with ADP-BeF_3_^−^ (pale green and blue), the magnesium ion (green) and coordinated water molecules (red). Both DEAH-box ATPases interact with the nucleotide in an identical manner. (*c*) Superposition of all structurally characterized DE*x*H-box ATPases bound to an ATP analog. The conformation of the helicase core in the ATP-bound state is highly conserved.

In order to investigate the discrepant manner of function of Prp2 in terms of unwinding, we crystallized Prp2 from *C. thermophilum* in the presence of ADP-BeF_3_^−^ and a U_12_-ssRNA, solved the crystallographic phase problem by means of molecular replacement and refined the structure at a resolution of 2.1 Å. The Prp2 construct used contains amino acids 286–921 and comprises the helicase core, composed of RecA1 and RecA2 domains, and the C-terminal domains, with winged-helix (WH), helix-bundle (HB) and oligonucleotide-binding (OB) domains (Fig. 1 ▸ *a*). The truncated N-terminal extension is only present as ten amino acids, as it has been proven that the helicase core and the C-terminal domains are the key domains for the ATPase function of the DEAH-box family (Tauchert *et al.*, 2017 ▸; Hamann *et al.*, 2019 ▸).

Seven of the 12 RNA nucleotides were traceable in the electron-density map (Supplementary Fig. S2*a*). For U_2_, only a model of the sugar-phosphate could be built. The ssRNA binds to an RNA-binding tunnel between the helicase core and the C-terminal domains, as previously reported for the spliceosomal DEAH-box ATPases Prp43 and Prp22 (Fig. 1 ▸ *a*; Tauchert *et al.*, 2017 ▸; Hamann *et al.*, 2019 ▸; He *et al.*, 2017 ▸). The ssRNA interacts mainly with its sugar-phosphate backbone *via* polar interactions with Prp2 and leads to sequence-nonspecific binding. The only exception is U_3_, where the base hydrogen-bonds to Gln516. RNA nucleotides U_4_–U_7_ are found in a stacked conformation and the backbone interacts with residues of the conserved sequence motifs (Ia, Arg352 and Arg353; Ib, Thr395; IV, Gln516; V, Thr572 and Asn573) and conserved structural features (hook-turn, Arg380; hook-loop, Ser547; β-hairpin, Lys594). These interactions are identical among structurally characterized spliceosomal DEAH-box ATPases and ensure a stack of four RNA nucleotides in the ATP-bound state and a stack of five RNA nucleotides in the adenosine nucleotide-free state (Tauchert *et al.*, 2017 ▸; Hamann *et al.*, 2019 ▸). The U_1_–U_3_ stretch is only stabilized by three polar interactions of the base of U_3_ with Gln516 and of the U_2_ phosphate with His877 and Thr900 (Fig. 1 ▸ *a*). The RNA molecule is not involved in any crystal contacts.

The ATP-mimic ADP-BeF_3_^−^ is sandwiched between the RecA-like domains (Fig. 1 ▸ *a*, Supplementary Fig. S2*b*). The conserved residues comprising the active site bind ADP-BeF_3_^−^ in a virtually identical manner to that seen in the ctPrp43–ADP-BeF_3_^−^ complex (I, Gly232, Gly325, Lys326, Thr327 and Thr328; Ia, Gln350 and Arg362; II, Asp418 and Glu419; V, Ser578; VI, Gln621, Arg625 and Arg628; Fig. 1 ▸ *b*; Tauchert *et al.*, 2017 ▸). This leads to an arrangement of the helicase core that is conserved among DE*x*H-box ATPases in the ATP-bound catalytic state (Fig. 1 ▸ *c*; Tauchert *et al.*, 2017 ▸; Prabu *et al.*, 2015 ▸; Chen *et al.*, 2018 ▸).

### 3.2. Putative exit channel for phosphate after ATP hydrolysis   

**Figure 2:**
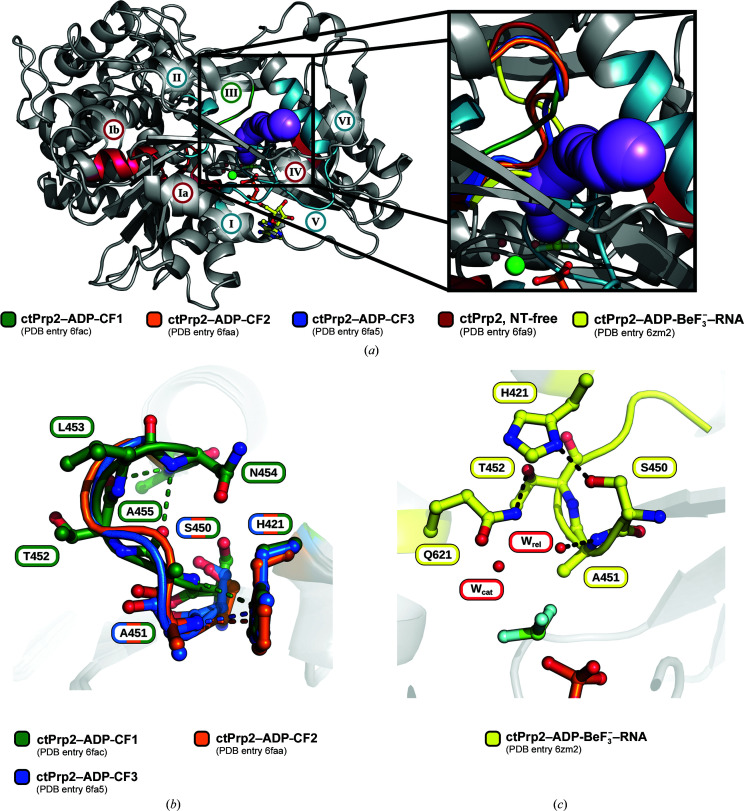

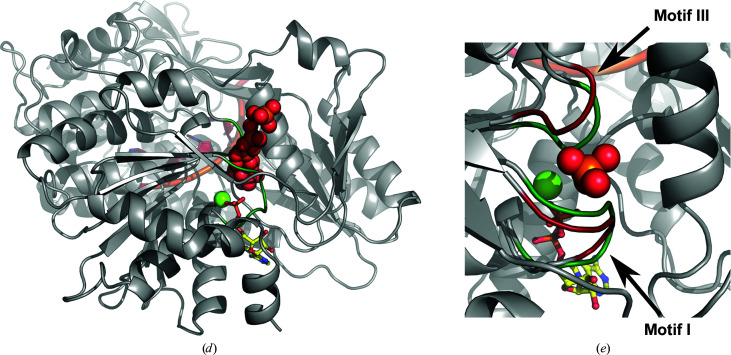
Movements of conserved sequence motif III control the formation of a channel connecting the nucleotide-binding site to the protein surface. (*a*) In the ctPrp2–ADP-CF1 structure, motif III adopts a conformation that allows the formation of a channel that connects the γ-phosphate position of the active site to the exterior of the protein. In other ADP- and ADP-BeF_3_^−^-bound structures motif III closes this channel. The exit channel is highlighted as purple spheres. CF stands for crystal form. (*b*) Overview of motif III interactions in the ADP-bound state. (*c*) Overview of motif III interactions in the ADP-BeF_3_^−^-bound state. (*d*) Exemplary trajectory of the γ-phosphate through the exit channel based on MD calculations. (*e*) Only minor movements of motifs I and III allow trespassing of the γ-phosphate through the exit channel (green, ATP-bound state; red, moved motifs after MD calculations).

By analyzing differences in the ψ and φ angles of all available ctPrp2 structures, the regions exhibiting the greatest deviations in conformation were identified (Supplementary Fig. S3). The flexibility of the β-hairpin and motif VI have already been discussed by Schmitt *et al.* (2018 ▸) and the role of the conformational variance of motif V has been described by Hamann *et al.* (2019 ▸) (Supplementary Fig. S3). Due to the rotation of the RecA2 domain between the ATP- and the ADP-bound states, the linker connecting the RecA-like domains also displays large differences in ψ and φ angles. Interestingly, motif III (SAT) also exhibits increased conformational variability, which has not been described before. This motif has been proposed to play a role in coupling ATP hydrolysis to unwinding (Schwer & Meszaros, 2000 ▸; Gross & Shuman, 1998 ▸; Heilek & Peterson, 1997 ▸; Pause & Sonenberg, 1992 ▸). It is located close to the phosphate moiety of the bound adenosine nucleotides, and crystal structures of Prp2 in complex with ADP or ADP-BeF_3_^−^ show that it is able to adopt three different conformations (Fig. 2 ▸ *a*; Supplementary Figs. S4*a*–S4*d*). Interestingly, in one of the ADP-bound structures (ctPrp2–ADP-CF1; green) it exhibits a conformation that opens a tunnel connecting the γ-phosphate position of the active site to the surface of the protein (Fig. 2 ▸ *a*; Supplementary Fig. S5). This tunnel could potentially represent an exit passage for the resulting inorganic phosphate after ATP hydrolysis that has not yet been described. All other motif III conformations in the ADP-bound state do not allow the formation of a tunnel that connects the active site to the surface. In all ADP-bound crystal structures His421 of the eponymous DEAH-motif (motif II) interacts via a hydrogen bond with the main-chain N atom of Ala451, but only in ctPrp2–ADP-CF1 does this alanine exhibit a conformation that ensures the start of an α-helix at this position (Fig. 2 ▸ *b*). This alternative conformation displaces Ala451, enabling the formation of the channel. In the ATP-bound state this channel is as well occluded by motif III (Fig. 2 ▸ *a*). Here, His421 of motif II interacts with the side chain of Ser450, and Gln621 of motif VI hydrogen-bonds to the side chain of Thr452 (Fig. 2 ▸ *c*). Additionally, the Ala451 main-chain N atom, which interacts with His421 in the ADP-bound state, is now involved in a hydrogen bond to the relay water of the active site (Dittrich & Schulten, 2005 ▸).

To test whether this tunnel indeed represents the most likely exit pathway for the γ-phosphate, we used all-atom molecular-dynamics (MD) simulations. Because the dissociation of DHP occurs on long time scales, we accelerated the dissociation using random-accelerated MD simulations (RAMD; Lüdemann *et al.*, 2000 ▸). RAMD is an established technique used to identify possible exit pathways for ligands. In RAMD, an additional force acting in a random direction is applied to the ligand, and the direction of the force is updated if the ligand cannot travel further in the current direction, implying that the ligand has reached a ‘dead end’. Here, by running many repeated RAMD simulations, we tested whether the γ-phosphate may exit Prp2 and Prp43 via one predominant or via multiple exit pathways.

We found that among 30 independent RAMD simulations with a successful dissociation event in the case of Prp2, DHP exited 24 times via a pathway between motifs I and III (Fig. 2 ▸ *d*). In only four out of 30 simulations, DHP exited Prp2 in the opposite direction via the ATP-binding site (Supplementary Fig. S7*a*). A similar pattern was observed in Prp43, where DHP exited through the suggested channel in 18 out of 25 simulations (Supplementary Fig. S7*b*). Here, four simulations also showed an alternative pathway out of the enzyme similar to that in Supplementary Fig. S7*a* (Supplementary Fig. S7*c*). These findings suggest that the pathway between motifs I and III exhibits the lowest free-energy barrier, whereas other putative pathways would require larger, energetically more unfavorable structural rearrangements. Indeed, visual inspection of the trajectories showed that minor fluctuations of motifs I and III are sufficient to allow the dissociation of DHP (Fig. 2 ▸ *e*). Taken together, the simulations strongly support release of the γ-phosphate prior to dissociation of ADP in Prp2 and Prp43, and this might also be conserved in other family members.

### 3.3. Divergent 5′ RNA conformations   

**Figure 3:**
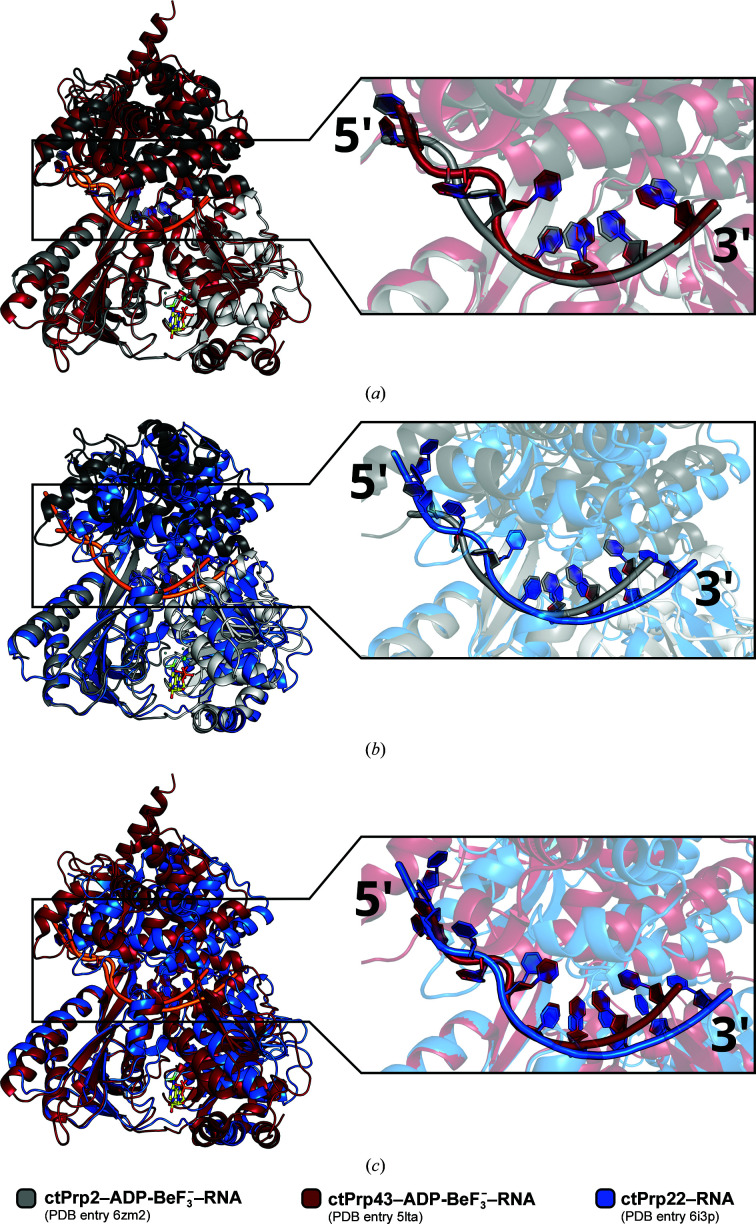
Comparison of ssRNA binding to spliceosomal DEAH-box ATPases. All ssRNAs bound to spliceosomal DEAH-box ATPases exhibit a kink in the 5′ region. When the RecA2 domains are superimposed [Prp2/Prp43 in (*a*), Prp2/Prp22 in (*b*) and Prp43/Prp22 in (*c*)], the kinks in the Prp43 and Prp22 structures share a similar position and only the kink in the Prp2 structure is differently positioned.

In all crystal structures of RNA-bound spliceosomal DEAH-box ATPases the 3′ region of the ssRNA exhibits a stacked conformation that is stabilized by conserved inter­actions with the conserved sequence motifs of both RecA-like domains (Tauchert *et al.*, 2017 ▸; Hamann *et al.*, 2019 ▸; He *et al.*, 2017 ▸). The stack accommodates either four or five RNA nucleotides, depending on the adenosine nucleotide state, and extends from the 3′ end of the RNA to the β-hairpin of the RecA2 domain. This structural feature ends the stack and redirects the 5′ region of the RNA through the RNA-binding tunnel. While the stacking of the 3′ region seems to be conserved in terms of conformation, the 5′ region shows different conformations in RNA-bound DE*x*H-box ATPase crystal structures (Hamann *et al.*, 2019 ▸). Interestingly, all spliceosomal DEAH-box ATPases exhibit a kink in the 5′ region, but a superposition of the RecA2 domains reveals differences in the position of this kink in Prp2 (Figs. 3 ▸ *a* and 3 ▸ ▸ *b*). In the Prp2 crystal structure the kink is introduced significantly closer to the 5′ end when compared with the Prp43 or Prp22 structures, whereas it roughly overlaps when directly comparing the Prp43 and Prp22 structures (Fig. 3 ▸ *c*). An analysis of the electrostatic potential of the DEAH-box ATPases shows that Prp2 is significantly more positively charged than Prp43 and Prp22 in the region of the RNA kink (Supplementary Fig. S8). This positively charged patch on Prp2 might contribute to a divergent guiding of the RNA backbone into an alternative 5′ conformation with a shifted kink.

### 3.4. A loop in the C-terminal domain threads the 5′ RNA region through a tunnel in the ATP-bound state   

**Figure 4:**
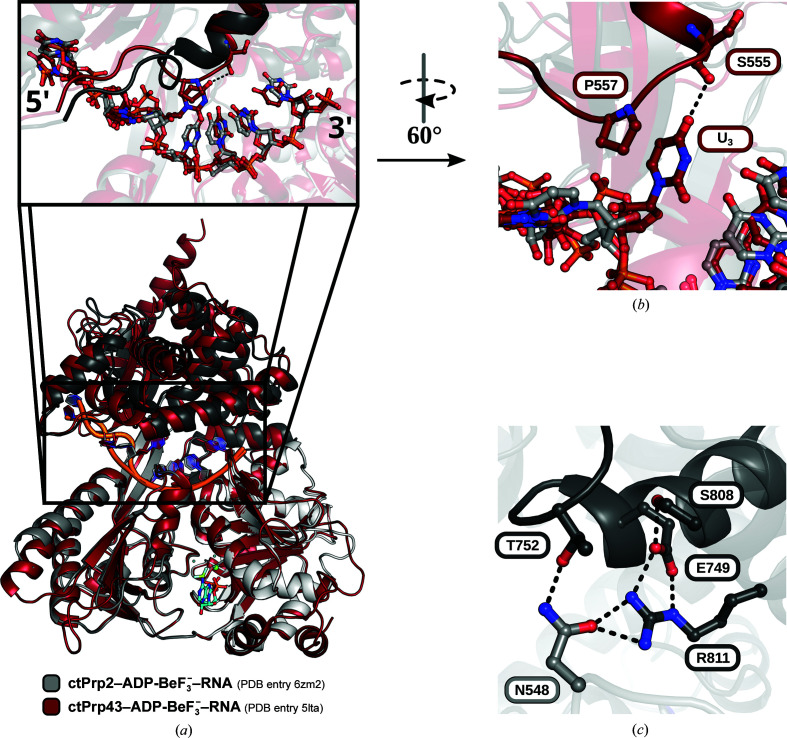
The C-terminal loop dictates the conformation of the 5′ RNA region. (*a*) Superposition of Prp2 and Prp43 in the pre-catalytic state via the helicase core. The 3′ stacked RNA region superposes almost identically, but at the beginning of the 5′ region the RNA bound to Prp43 interacts with the C-terminal loop. This loop has a different conformation in Prp2 and does not interact with the RNA. (*b*) The base of the first nucleotide of the 5′ RNA region interacts with a proline and a serine of the Prp43 C-terminal loop. (*c*) The alternative conformation of the Prp2 C-terminal loop is stabilized by interactions with surrounding residues belonging to the helix-bundle domain and the hook-loop of the RecA2 domain.

Prp43 is the only genuine DEAH-box ATPase with published structures in the ATP- and RNA-bound state (Tauchert *et al.*, 2017 ▸; He *et al.*, 2017 ▸). A superposition of the presented Prp2 structure with the ADP-BeF_3_^−^- and RNA-bound ctPrp43 structure highlights the virtually identical conformation of the helicase core and the highly similar position of the C-terminal domains (Fig. 4 ▸ *a*). The stacked 3′ RNA region also superposes well, but at the first RNA nucleotide position after the stack the path of the RNA differs. In the ctPrp43 structure a kink in the RNA backbone is introduced at this position, mainly by the stacking of U_3_ with Pro557 and a hydrogen bond to Ser555 (Fig. 4 ▸ *b*). These two residues are part of a loop in the helix-bundle domain, part of the C-terminal domains (Leu554–Gln558). The corresponding loop in ctPrp2 (Leu747–Thr752) displays an alternative conformation with a more extended α-helix due to an insertion. This conformation is stabilized by a network of polar interactions with surrounding residues (Fig. 4 ▸ *c*). Thr752 of this C-terminal loop hydrogen-bonds to Asn548 from the hook-loop motif of the RecA2 domain (Tyr546–Asn548), which itself interacts with Arg811 from the helix-bundle domain. Arg811 also interacts with Glu749, which is stabilized by a hydrogen bond to Ser808. All of these interactions lock the C-terminal loop in a conformation that is incompatible with an RNA kink as seen in the ctPrp43 structure. Instead, a kink in the RNA backbone is introduced at a position closer to the 5′ end. While the conformation of the stacked 3′ RNA region is highly conserved, the divergent conformations of the 5′ RNA region seem to be highly influenced by the C-terminal loop.

### 3.5. Conservation of the C-terminal loop   

**Figure 5:**
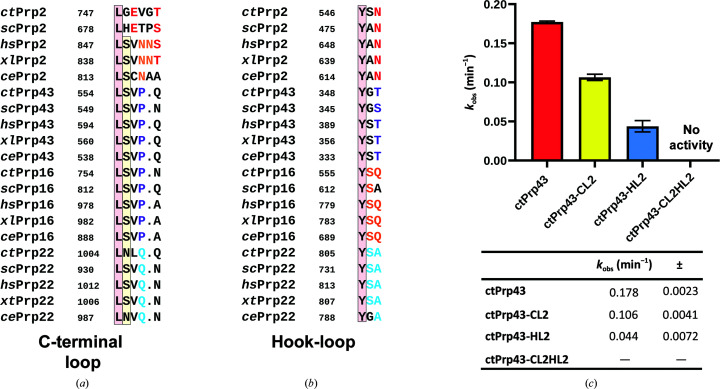
Sequence conservation of the C-terminal loop and hook-loop. Sequence alignment of the C-terminal loop (*a*) and the hook-loop (*b*) of Prp2, Prp43, Prp16 and Prp22 in *Chaetomium thermophilum*, *Saccharomyces cerevisiae*, *Homo sapiens*, *Xenopus laevis* and *Caenorhabditis elegans*. (*c*) Helicase activities of various ctPrp43 constructs with a mutated C-terminal loop or/and hook-loop. An overview of the activities is shown as a bar plot and the *k*_obs_ values are listed below. All experiments were performed in triplicate; the standard deviation is highlighted as error bars and indicated as ± in the table.

Sequence alignment of the C-terminal loop reveals that it differs among the four spliceosomal DEAH-box ATPases but is conserved in each individual ATPase among different organisms (Fig. 5 ▸ *a*). While Prp43 and Prp16 share a highly conserved proline that stacks with a base of the RNA in the ctPrp43–ADP-BeF_3_^−^–RNA structure, Prp22 has a conserved glutamine at this position. Prp2 is the only spliceosomal DEAH-box ATPase with an insertion in this loop, contributing to its unique conformation (Fig. 5 ▸ *a*; Supplementary Fig. S9). Interestingly, although an insertion is conserved, the type of insertion differs among higher eukaryotes (Supplementary Fig. S10*a*). In fungal proteins the glutamate and threonine of the C-terminal loop are conserved, while in some fungal members the threonine is replaced by a serine, which should still maintain the same interacting properties with the hook-loop as observed in the ctPrp2–ADP-BeF_3_^−^–RNA structure. In representatives from animals the glutamate is not present and two asparagine residues are instead conserved.

The asparagine of the hook-loop that interacts with the C-terminal loop in the ctPrp2–ADP-BeF_3_^−^–RNA structure is present in Prp2 from all analyzed organisms and suggests that the interplay between these two structural features is conserved in Prp2 (Fig. 5 ▸ *b*, Supplementary Fig. S10*b*).

The conserved insertion in the C-terminal loop, together with the interaction with the hook-loop, leads to a unique conformation of the C-terminal loop in Prp2, which might play a regulatory role (Supplementary Fig. S9). In contrast, the C-terminal loops of ctPrp43 and ctPrp22 show the same length of the α-helix as well as a very similar conformation of the loop itself.

### 3.6. The C-terminal loop and hook-loop act in concert to regulate helicase activity   

In order to test the role of the C-terminal loop in the helicase activity and its interplay with the hook-loop, we mutated these two motifs in Prp43 and analyzed the impact on the helicase activity. Therefore, all measurements were performed using a helicase assay previously established for ctPrp43 using double-stranded RNA with a 3′ overhang, a fivefold molar excess of the G-patch motif of ctPfa1 and 1 m*M* ATP (Tauchert *et al.*, 2017 ▸). Both motifs in ctPrp43 were mutated to the respective sequences in ctPrp2. The mutant with an exchanged C-terminal loop (ctPrp43-CL2; ^555^SVPQ^559^→^555^GEVGT^560^) showed a decreased helicase activity, with a *k*_obs_ of 0.106 min^−1^ compared with a *k*_obs_ of 0.178 min^−1^ for wild-type ctPrp43 (Fig. 5 ▸ *c*). The exchange of the hook-loop (ctPrp43-HL2; ^349^GT^350^→^349^SN^350^) had a more severe effect on the helicase activity, leading to a fourfold lower helicase activity (*k*_obs_ = 0.044 min^−1^) compared with ctPrp43. Most strikingly, when both motifs were mutated (ctPrp43-CL2HL2) no helicase activity could be detected, which strongly supports the idea that the motifs act in concert to regulate helicase activity.

All ctPrp43 mutants were also tested for ATPase activity in order to verify their functional integrity (Supplementary Fig. S11). All constructs exhibited a similar stimulation pattern, showing stimulation by the ctPfa1 G-patch motif, which was even stronger in the presence of ssRNA.

## 4. Discussion   

Among the spliceosomal DEAH-box ATPases, Prp2 plays a special role as to date no dsRNA-unwinding activity could be determined for this ATPase (Warkocki *et al.*, 2015 ▸; Bao *et al.*, 2017 ▸; Kim *et al.*, 1992 ▸). In contrast, for Prp43, Prp22 and Prp16 *in vitro* helicase activity has been characterized (Tauchert *et al.*, 2017 ▸; Tanaka *et al.*, 2007 ▸; Christian *et al.*, 2014 ▸; Schwer & Gross, 1998 ▸; Tanaka & Schwer, 2005 ▸; Wang *et al.*, 1998 ▸). This raises the question as to why Prp2 functions so differently despite its sequence and structural similarity to the other spliceosomal DEAH-box ATPases. The elucidation of the determinant that impedes Prp2 from being a competent unwindase might also provide additional insights into the regulatory aspects of DEAH-box ATPases capable of disrupting duplex RNAs. In order to address these questions, we solved the crystal structure of ctPrp2 with bound U_7_-RNA and the ATP analog ADP-BeF_3_^−^ (Fig. 1 ▸ *a*).

By comparing the ATP-bound state of ctPrp2 with previously published ADP-bound structures, we were able to identify significant conformational changes of motif III between these states. This motif exhibits a closed conformation in the presence of ADP-BeF_3_^−^, which is stabilized by polar contacts with the neighboring motifs II and VI (Fig. 2 ▸ *c*). It additionally interacts with the relay water of the adenosine nucleotide-binding site (Supplementary Fig. S2*f*). Active-site water molecules are structurally highly conserved and specific to each nucleotide-bound state. In the ADP-bound state four water molecules are always coordinated by the active-site magnesium, whereas in the ATP-bound state it coordinates three water molecules (Schmitt *et al.*, 2018 ▸; Tauchert *et al.*, 2016 ▸, 2017 ▸; Walbott *et al.*, 2010 ▸; He *et al.*, 2010 ▸). The ATP-bound state additionally harbors a catalytic water molecule and a relay water molecule that are present in all structures of this state (Dittrich & Schulten, 2005 ▸). The magnesium-coordinated water molecules, as well as the catalytic water molecule, have been proposed to play a crucial role in dictating the position of the RecA2 domain (Hamann *et al.*, 2019 ▸; Supplementary Figs. S2*c*–S2*e*). Due to the fact that these water molecules are specific to a certain catalytic state, a sensor serine in RecA2 motif V is able to discriminate between the ADP-bound and ATP-bound states in order to accordingly position the RecA2 domain. The relay water molecule might also function as an active-site component that is sensed by motif III in order to induce the closed conformation of this motif. Conversely, this conformation might be needed to properly position the relay water for its role during ATP hydrolysis. Interestingly, one ADP-bound Prp2 structure (PDB entry 6fac; Schmitt *et al.*, 2018 ▸) exhibits an open conformation of motif III, which results in a direct connection of the nucleotide-binding site to the surface of the protein (Fig. 2 ▸ *a*, Supplementary Fig. S5). To date, the exact order of events after the generation of ADP and P_i_ is still unknown, and it is not clear which is released first. For some DEAD-box helicases it has been shown that P_i_ is released prior to ADP (Wong *et al.*, 2016 ▸). In the case that P_i_ is also released first in the DEAH-box ATPase family, another exit passage apart from the ATP-entry/ADP-exit site would be needed. The ctPrp2–ADP-CF1 structure shows the first evidence for such a channel connecting the phosphate-bound end of the active site to the outer surface, thereby providing an alternative passage that would not be in conflict with the bound ADP, which is strongly supported by MD simulations. All residues involved in formation of the exit channel (Ser450–Thr452 from motif III, His421 from motif II and Gln621 from motif VI) are identical in all DEAH-box ATPases (Supplementary Fig. S6). In fact, all of these residues are conserved among DEAH-box, NS3/NPH-II and Ski2-like ATPases. Only in the SF2 helicase subfamily of DEAD-box helicases do the key residues from motifs II and VI differ. Here, Gln621 is replaced by a histidine and His421 of the eponymous DEAH motif is replaced by an aspartate, virtually swapping charges. Interestingly, DEAH-box, NS3/NPH-II and Ski2-like ATPases all possess C-terminal domains which restrict the freedom of movement of the RecA domains in comparison to DEAD-box helicases. This difference in domain dynamics might require divergent ATP-hydrolysis mechanisms, which might be orchestrated by different key residues such as those involved in formation of the exit channel. In order to determine the exact order of events during ATP hydrolysis in DEAH-box ATPases, detailed biochemical analyses, as performed for the DEAD-box helicase Dbp5, could provide further insights (Wong *et al.*, 2016 ▸).

Although recent structural and biochemical evidence suggest that the spliceosomal DEAH-box ATPases might not need unwinding activity to fulfill their functions during splicing, Prp16, Prp22 and Prp43 nevertheless show *in vitro* helicase activity (Tauchert *et al.*, 2017 ▸; Tanaka *et al.*, 2007 ▸; Christian *et al.*, 2014 ▸; Schwer & Gross, 1998 ▸; Tanaka & Schwer, 2005 ▸; Wang *et al.*, 1998 ▸). Prp2 is the only spliceosomal DEAH-box ATPase for which no *in vitro* unwinding activity could be determined (Bao *et al.*, 2017 ▸; Kim *et al.*, 1992 ▸). Since a divergent mode of interaction of Prp2 with RNA might lead to the lack of unwinding activity, we crystallized Prp2 in the presence of ssRNA and the ATP analog ADP-BeF_3_^−^. A comparison with previously published RNA-bound structures of spliceosomal DEAH-box ATPases shows that while all exhibit a kink of the RNA in the 5′ region, it is shifted in the Prp2 structure compared with those of Prp43 and Prp22 (Fig. 3 ▸). On the one hand different electrostatic potentials of the ATPases close to this kink might influence the binding, while on the other hand a loop in the helix-bundle domain seems to play a crucial role in dictating the conformation of the RNA immediately after the interruption of the stack by the β-hairpin (Fig. 4 ▸; Supplementary Fig. S8; Tauchert *et al.*, 2017 ▸; Hamann *et al.*, 2019 ▸). Due to the strong dependence of the catalytic state on the overall conformation of DEAH-box ATPases, we compared the ADP-BeF_3_^−^- and RNA-bound structure of Prp2 with the structure of Prp43 in the same state. While the 3′ stacked region of the RNA superimposes almost identically, the conformation of the 5′ region shows major differences (Fig. 4 ▸ *a*). In the Prp43 structure the RNA is guided by interaction with a proline and a serine of a loop protruding out of the C-terminal domains (Figs. 4 ▸ *a* and 4 ▸ *b*). This interaction ensures the kink in the RNA directly after the β-hairpin. Prp2 lacks such an interaction with the RNA at this position, and instead this loop harbors a conserved insertion that ensures an alternative conformation of this loop, which is stabilized by multiple interactions with neighboring residues (Fig. 4 ▸ *c*). One of these interactions is with Asn548, which is part of the hook-loop (Prabu *et al.*, 2015 ▸). Interestingly, this structural feature has been proposed to play an important role in processivity in the DE*x*H-box helicase MLE. However, it has also been shown that the hook-loop is dispensable for helicase function in Prp43 (Tauchert *et al.*, 2017 ▸). The hook-loop and the C-terminal loop are in close proximity and might play similar roles in threading the RNA through the RNA-binding tunnel in the 5′ region. Since the RNA in Prp43 primarily interacts with the C-terminal loop in the 5′ region, the interaction with the hook-loop is not relevant. The hook-loop as well as the C-terminal loop show only low sequence conservation among the four spliceosomal DEAH-box ATPases, but are conserved in each DEAH-box ATPase (Figs. 5 ▸ *a* and 5 ▸ *b*). This suggests that the different spliceosomal DEAH-box ATPases have different conserved ways of threading the RNA through the tunnel after the stacked 3′ region. Prp43 mainly utilizes a conserved proline and serine of the C-terminal loop and exhibits short residues in the hook-loop that do not intervene in the binding. This explains why mutating the residues of the Prp43 hook-loop to similarly short glycine residues does not have any influence on the helicase activity (Tauchert *et al.*, 2017 ▸). Although Prp16 has a highly similar C-terminal loop, the hook-loop has a different property and might induce a different RNA conformation compared with Prp43. Prp22 has an unique glutamine instead of a proline in the C-terminal loop and the sequence of the hook-loop also differs from the others. Unfortunately, only an RNA-bound but nucleotide-free structure of Prp22 is available, in which the RecA2 domain is shifted and therefore the C-terminal loop and hook-loop are apart (Hamann *et al.*, 2019 ▸). In this structure these two structural features do not interact with each other and the C-terminal loop does not contact the RNA. The overall conformation of the C-terminal loop in Prp22 closely resembles that in Prp43 (Supplementary Fig. S9). Interestingly, in the structure of RNA- and ADP-BeF_3_^−^-bound Prp2, the C-terminal loop displays a distinct conformation due to an insertion. Instead of interacting with the RNA, it interacts with a conserved asparagine of the hook-loop and other surrounding residues (Fig. 4 ▸ *c*). This interaction network is likely to be conserved in Prp2 from different species and threads the 5′ RNA region in a completely different manner compared with Prp43 and Prp22 (Fig. 3 ▸). Interestingly, the conservation of the C-terminal loop shows major differences in sequence between representatives from fungi and animals; however, in both cases an insertion seems to be conserved as a feature of this structural motif in Prp2 (Supplementary Fig. S10*a*). Despite this difference in the sequence of the C-terminal loop, the hook-loop is consistently conserved among fungi and animals, suggesting a similar conformation and interaction network in animal Prp2s as seen for *C. thermophilum* Prp2 as a candidate from the fungi (Supplementary Fig. S10*b*).

In order to verify the importance of the C-terminal loop and its potential interplay with the hook-loop, we swapped these motifs from ctPrp43 with the respective motifs from ctPrp2. By using a fluorescence-based dsRNA-unwinding assay that has been previously established for ctPrp43, we were able to analyze the impact of these mutations on the helicase activity (Tauchert *et al.*, 2017 ▸). When exchanging the motifs separately a significant decrease in the unwinding capability of ctPrp43 could be observed, leading to a fourfold lower *k*_obs_ in the case of ctPrp43-HL2 (Fig. 5 ▸ *c*). This drastic loss in activity of the hook-loop mutant is particularly interesting, as the complete replacement of this motif (YGT) by glycines (GGG) has been shown to have no effect on helicase activity (Tauchert *et al.*, 2017 ▸). These findings together suggest that the hook-loop is only dispensable as long as the residues of this motif exhibit only short side chains. In ctPrp43-HL2 (YGT→YSN) the tyrosine is unchanged and the central glycine is replaced by a serine. While this serine in the Prp43 mutant is larger than the native glycine in *C. thermophilum*, Prp43s from other organisms also harbor a serine at this position, suggesting that a serine at this position might not impact the function of the motif (Fig. 5 ▸ *b*). The properties of the third position of this motif (ctPrp43, Thr; ctPrp2, Asn) is likely to have the most significant effect. While threonine and asparagine share the property of being polar, they differ in size. This position of the hook-loop is the closest to the C-terminal loop and in ctPrp2 the larger size of Asn548 allows contact of both motifs, which is not possible in ctPrp43 due to the smaller size of Thr350 (Fig. 4 ▸ *c*). Thus, in ctPrp43-HL2 the larger asparagine replacing the threonine might interfere with the C-terminal loop or the nearby base which stacks with the C-terminal loop proline, thereby altering the threading of the RNA in this position and leading to an impaired helicase capability of this mutant (Supplementary Fig. S12). This indicates that in Prp43 the C-terminal loop is the primary factor in properly positioning the RNA after the 3′ stacked region by introducing the kinked conformation at the beginning of the 5′ region, and the residues of the hook-loop are kept short in order not to intervene in this function. In contrast, in Prp2 the larger size of the asparagine of the hook-loop and the alternative conformation of the C-terminal loop due to an insertion ensure direct interaction of these two motifs (Fig. 4 ▸ *c*). These interactions seem to lock Prp2 in a conformation in which it is unable to interact with the RNA in this position in a comparable manner as seen in Prp43 and might therefore impede Prp2 from having helicase activity. In fact, a ctPrp43 mutant with both motifs swapped completely loses its ability to unwind dsRNA (Fig. 5 ▸ *c*). Since the individual exchanges only partially impaired the helicase activity of ctPrp43 and both exchanges together completely abolish its unwinding function, it can be assumed that the interplay of both motifs is required and sufficient to effectively impair the duplex-unwinding capability of a DEAH-box ATPase.

While Prp2 and Prp43 might represent two extreme examples, in which in one case the interplay between the C-terminal loop and hook-loop is required to impede function and in the other case interplay is avoided in order to guarantee function, it is imaginable that these two motifs regulate the other two spliceosomal DEAH-box ATPases, Prp16 and Prp22, in a different manner. For example, Prp16 has a highly similar C-terminal loop compared with Prp43, but an even larger polar residue (glutamine) in the hook-loop (Figs. 5 ▸ *a* and 5 ▸ *b*). Here, it is feasible that the hook-loop also interacts with the C-terminal loop as seen for Prp2, but due to the different properties of the C-terminal loop, which closely resembles that in Prp43, the interaction might not totally abolish the helicase function but might instead regulate it in a different manner, which was also the case for ctPrp43-HL2 (Fig. 5 ▸ *c*). Prp22, for example, also exhibits rather short hook-loop residues, but instead of a proline the C-terminal loop harbors a considerably larger glutamine, which could enable an interaction with the hook-loop (Figs. 5 ▸ *a* and 5 ▸ *b*). Such a divergent interplay could also lead to a different regulation of the helicase activity.

All known DE*x*H-box ATPases seem to rely on translocation for their functions, and thus the interaction and conformation of the 3′ stacked region of the bound ssRNA is highly conserved in all solved crystal structures of these members, as this region has been shown to be crucial for translocation function (Hamann *et al.*, 2019 ▸). In contrast, the interactions and conformations of the 5′ regions in ssRNA-bound structures differ significantly (Supplementary Fig. S13). While all of them are likely to need to maintain the basic ability to translocate, their targets and biological functions are highly diverse and the divergent modes of interaction with the 5′ region might be key to regulating these different tasks. DHX36, for example, unfolds G-quadruplexes at the 5′ end of an ssRNA with the help of its N-terminal extension, which wraps around the protein in order to reach the RNA-binding tunnel at the 5′ region of the ssRNA (Chen *et al.*, 2018 ▸). The DEAH-box ATPases Prp43, Prp22 and Prp16 have all been reported to be able to unwind dsRNAs, but the interaction with the 5′ region differs between the structurally characterized Prp43 and Prp22 (Tauchert *et al.*, 2017 ▸; Hamann *et al.*, 2019 ▸). Prp43 interacts primarily with this region via its C-terminal loop in the helix-bundle domain, while Prp22 mainly uses a stacking triad in the OB domain to interact with the 5′ region, which is not present in Prp43. These differences are likely to fine-tune their functions in order to adapt to their specific tasks. Finally, the most severe impact of this 5′-region regulation can be observed in Prp2, leading to complete impairment of the unwinding function.

## Supplementary Material

PDB reference: Prp2, complex with RNA and ADP-BeF_3_^−^, 6zm2

Supplementary Figures. DOI: 10.1107/S2059798321001194/ud5019sup1.pdf

## Figures and Tables

**Table 1:** Data-collection and refinement statistics for ctPrp2–ADP-BeF_3_^−^–RNA

Data collection	
Space group	*P*2_1_2_1_2_1_
*a*, *b*, *c* (Å)	48.7, 100.4, 141.0
X-ray source	P14, PETRA III, DESY
Oscillation range (°)	0.1
Wavelength (Å)	0.9793
Resolution range (Å)	81.78–2.10 (2.20–2.10)
No. of observed reflections	561018 (72672)
No. of unique reflections	41019 (5228)
Completeness (%)	99.5 (99.0)
*R*_meas_ (%)	13.1 (132.6)
Average *I*/σ(*I*)	14.88 (2.21)
Multiplicity	13.68 (13.90)
CC_1/2_ (%)	99.9 (81.3)
Wilson *B* factor (Å^2^)	42.30
Refinement	
Resolution (Å)	81.78–2.10 (2.15–2.10)
No. of reflections	41000
*R*_work_ (%)	18.52 (25.70)
*R*_free_ (%)	23.31 (27.90)
Total No. of atoms	5355
Protein residues	627
Water molecules	195
R.m.s. deviations	
Bond lengths (Å)	0.008
Bond angles (°)	0.818
Mean *B* factors (Å^2^)	
Protein	44.65
RNA	68.43
ADP-BeF_3_^−^	33.17
Water	48.38
Ramachandran statistics	
Favored (%)	97.58
Allowed (%)	2.26
Outliers (%)	0.16
*MolProbity* clashscore	4.66
PDB code	6zm2
