# Supplementary material for: The structure of Prp2 bound to RNA and ADP-BeF3 − reveals structural features important for RNA unwinding by DEAH-box ATPases
Source: Acta Crystallogr D Struct Biol. 2021 Mar 30;77(Pt 4):496–509. doi: 10.1107/S2059798321001194 (PMC8025883; doi:10.1107/S2059798321001194)
Supplement: Supplementary file 1 [file d-77-00496-sup1.pdf]

STRUCTURAL
BIOLOGY

Volume 77 (2021)

Supporting information for article:

The structure of Prp2 bound to RNA and ADP-BeF$_3^-$ reveals structural features important for RNA unwinding

Florian Hamann, Lars C. Zimmerningkat, Robert A. Becker, Tim B. Garbers, Piotr Neumann, Jochen S. Hub and Ralf Ficner

SUPPLEMENTAL INFORMATION

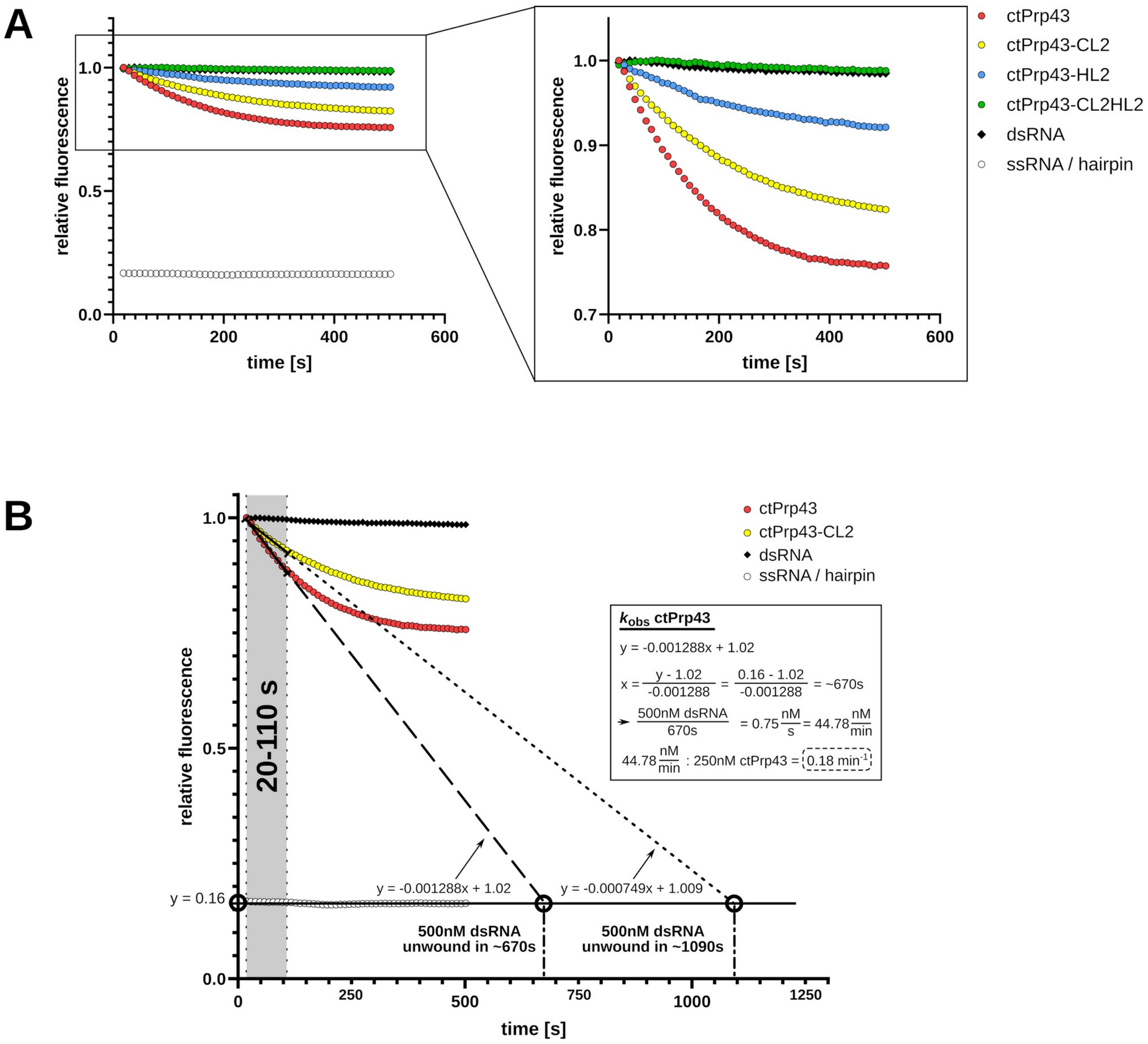

S1: Exemplary raw helicase activity data of ctPrp43 mutants and calculation of k_{obs}. (A) Typical curve progressions for the fluorescence-based helicase assay of an individual measurement of each mutant (red: ctPrp43; yellow: ctPrp43-CL2; blue: ctPrp43-HL2; green: ctPrp43-CL2HL2). All mutants were measured in triplicates. The fluorescence signal for dsRNA (black) and unwound ssRNA forming a hairpin (white) is shown. **(B)** Exemplary calculations of k_{obs} for ctPrp43 und ctPrp43-CL2.

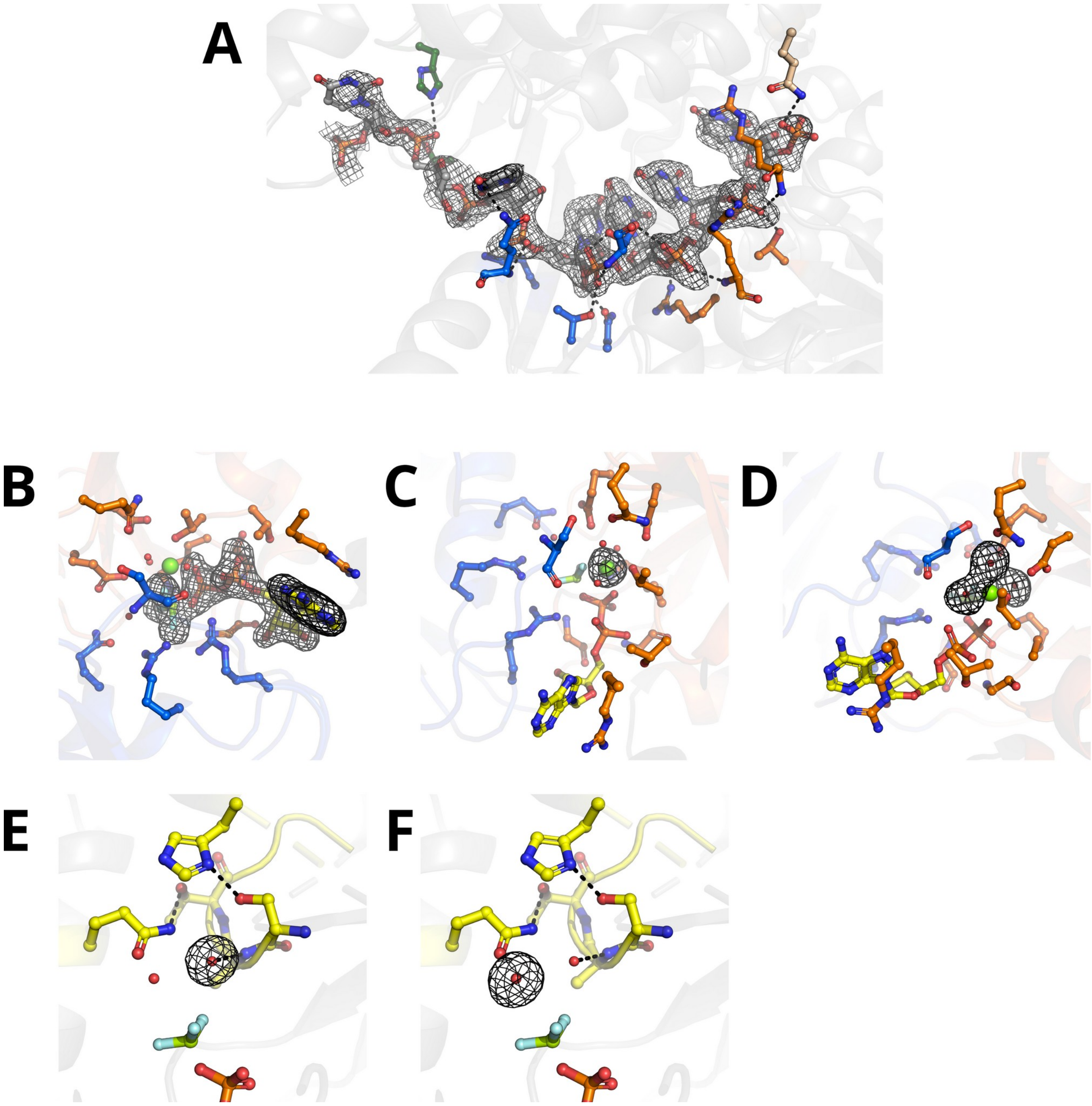

S2: Ligands bound to ctPrp2 in the complex structure with ADP-BeF$_3^-$ and RNA. mF_o-DF_c electron-density omit maps of poly-U ssRNA **(A)**, ADP-BeF$_3^-$ **(B)**, magnesium **(C)**, water molecules coordinated by magnesium **(D)**, relay water **(E)** and catalytic water **(F)** are displayed as black meshes at 3σ.

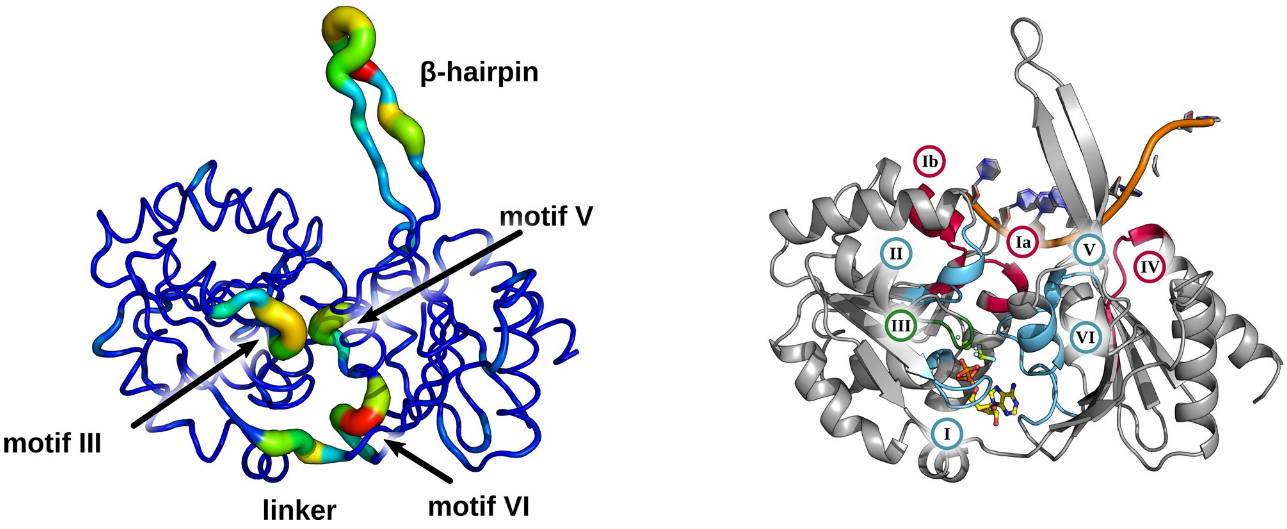

S3: Overview of sequence and structure motifs movements between different catalytic states. Differences of psi and phi angles for single residues of the helicase core of all available Prp2 structures are plotted onto the Prp2 structure. Motifs III,V and VI as well as the β-hairpin and the linker connecting both RecA-like domains show movements of the mainchain. An overview of conserved sequence motifs spread over the two RecA-like domains is given in the right panel.

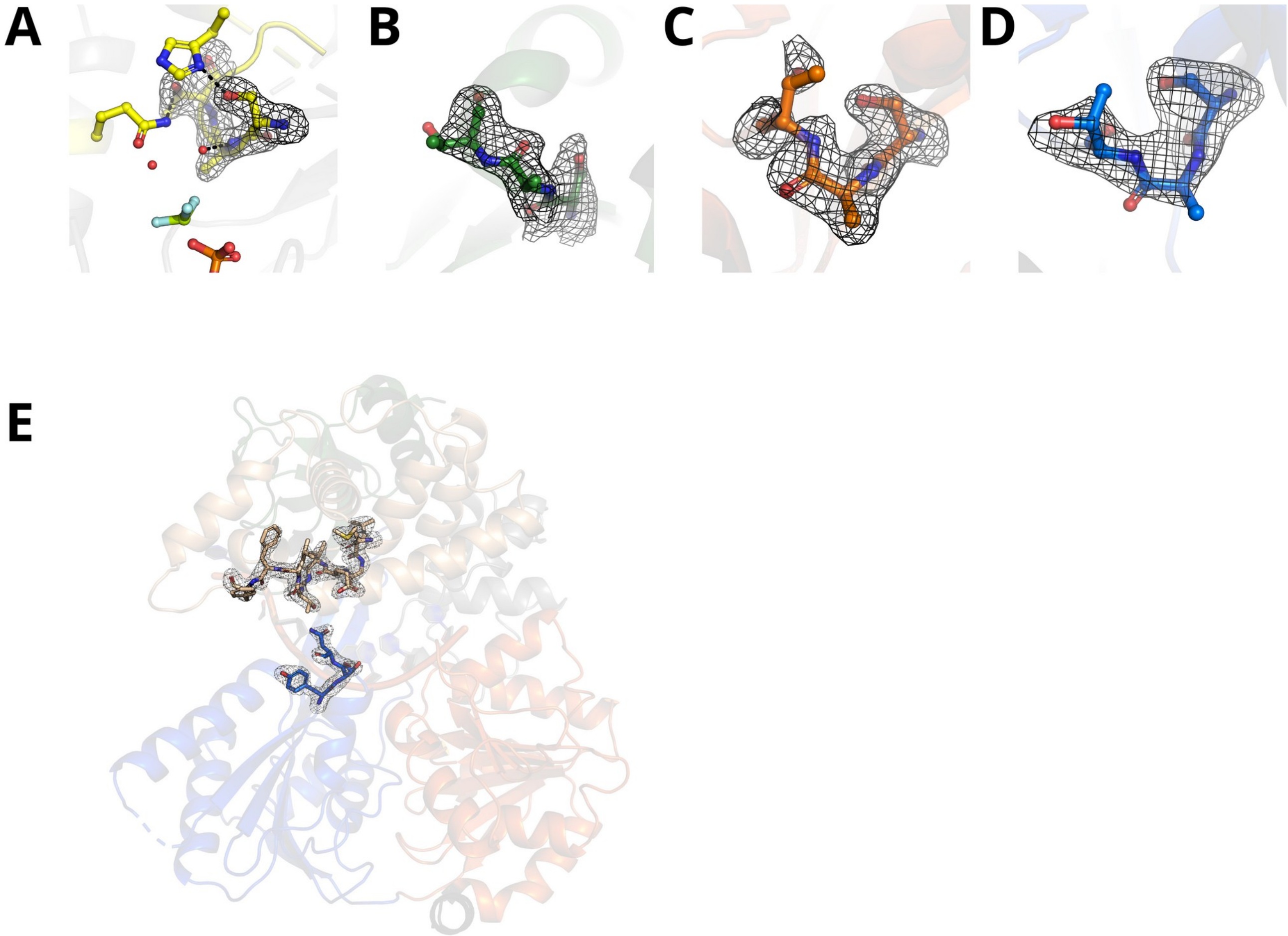

S4: Omit maps of motif III, C-terminal loop and hook-loop. mF_o-DF_c electron-density omit maps of motif III from ADP-bound ctPrp2 crystal structures CF1 (PDBid: 6fac) **(A)**, CF2 (PDBid: 6faa) **(B)** and CF3 (PDBid: 6fa5) **(C)** as well as motif III **(D)**, C-terminal loop and hook-loop **(E)** from the RNA- and ADP-BeF3-bound structure are displayed as black meshes at 3σ.

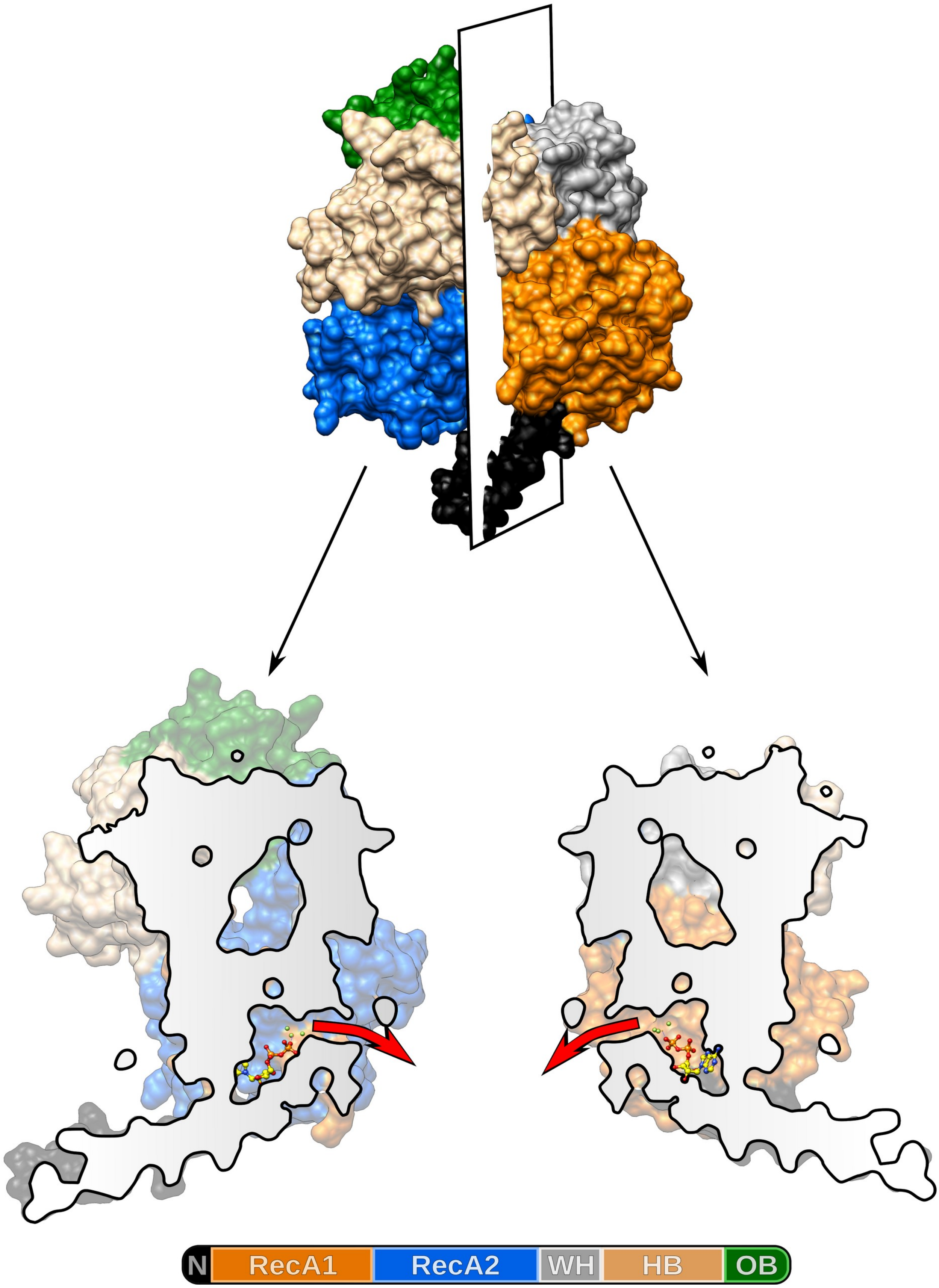

S5: Cross section of Prp2 with bound ADP (PDBid: 6fac). A cross section of one of the ADP-bound structures of Prp2 reveals a direct connection of γ-phosphate binding site with protein surface. This channel might serve as an exit passage for the γ-phosphate after ATP hydrolysis. The ADP-$BeF3^-$ from the ATP-bound Prp2 structure was modeled into the active site of the ADP-bound Prp2 structure in order to visualize the close position of the γ-phosphate to the channel. The ADP moiety is depicted as balls and sticks and the $BeF3^-$ is shown as green spheres. The surface representation of Prp2 is colored as described in Figure 1.

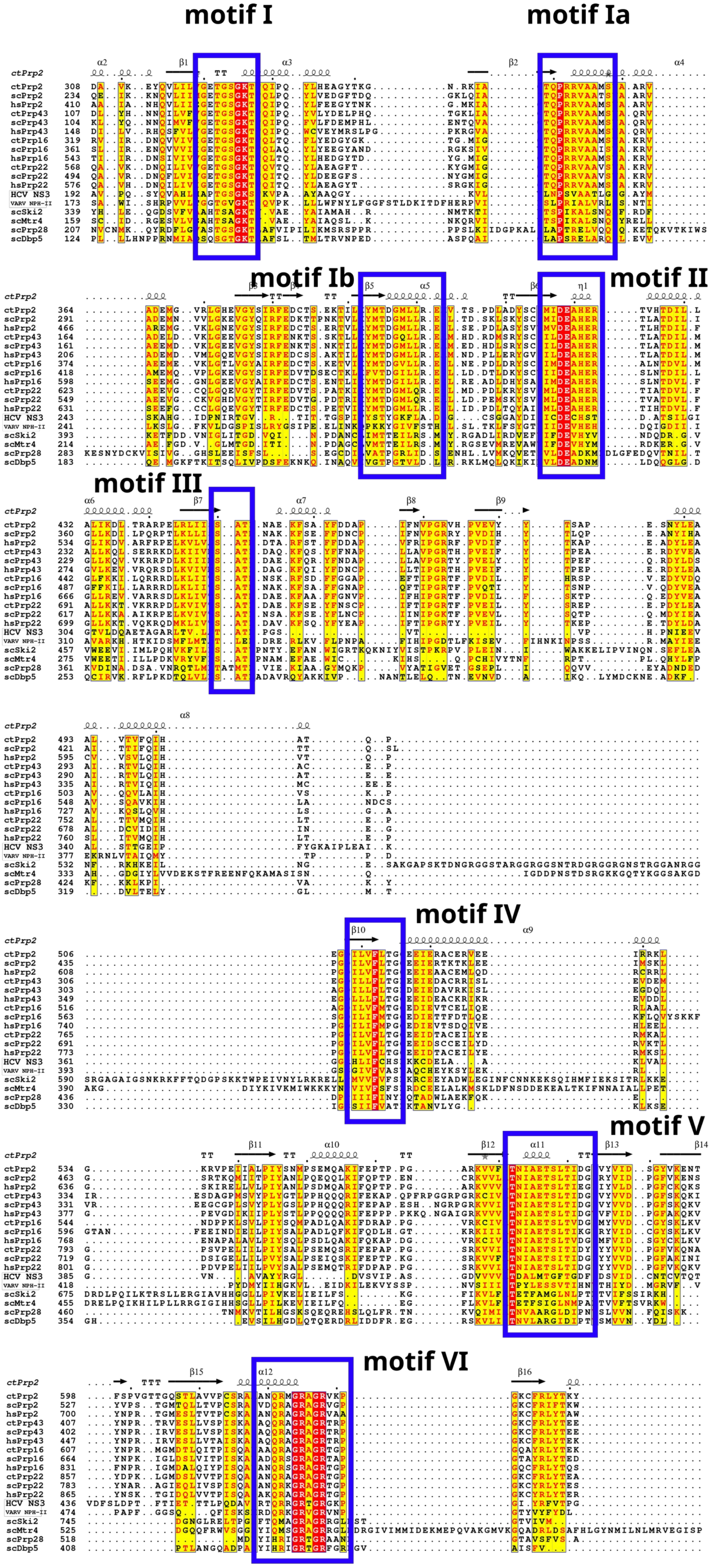

S6: Sequence alignment of SF2 helicases. Sequence alignment of DEAH-box ATPases (Prp2, Prp43, Prp16 and Prp22), NS3/NPH-II helicases (hepatitis C virus [HCV] NS3 and variola virus [VARV] NPH-II), Ski2-like helicases (Ski2 and Mtr4) and DEAD-box ATPases (Prp28 and Dbp5). Conserved sequence motifs are highlighted in blue boxes and labeled accordingly.

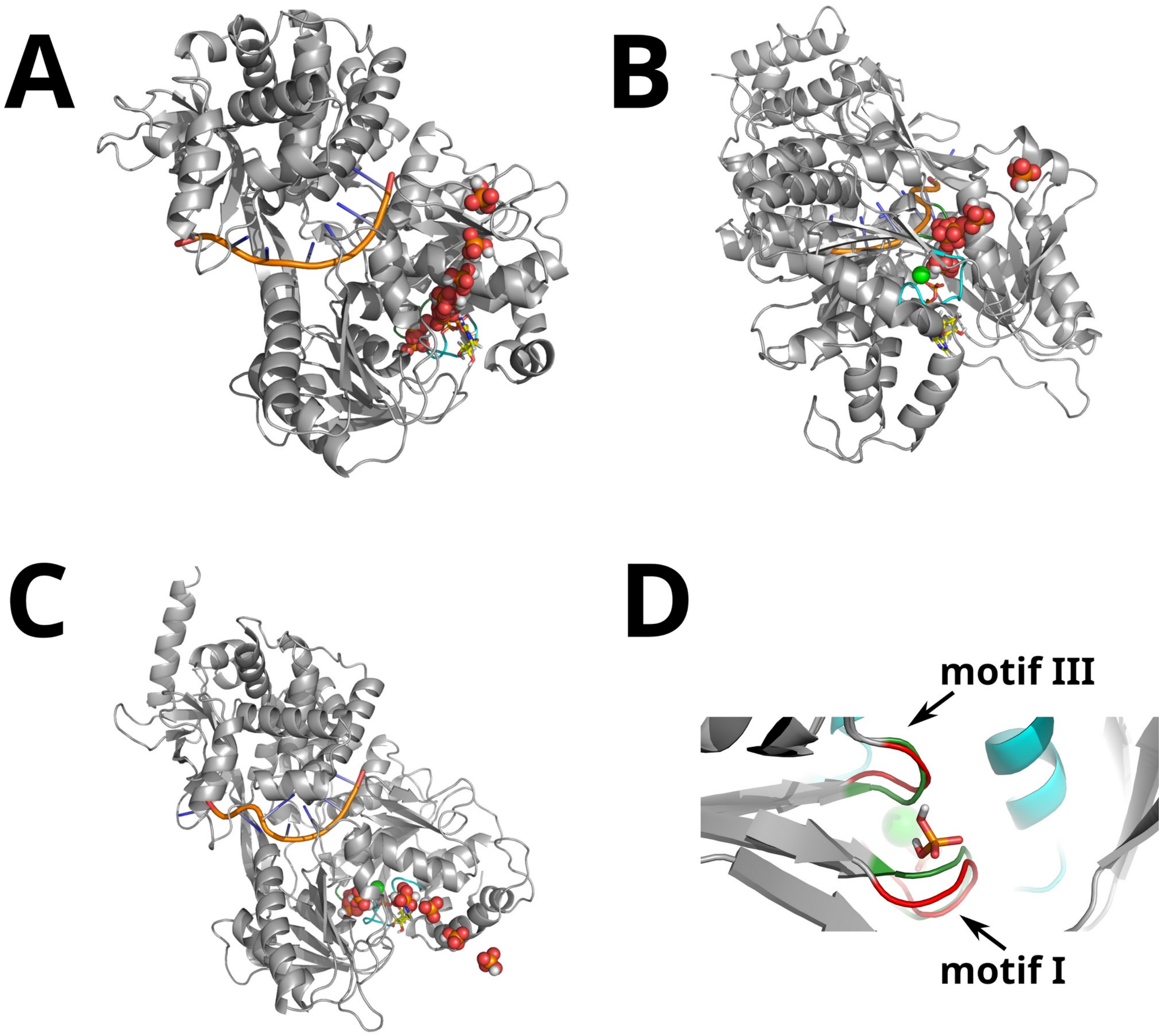

S7: MD simulations of γ-phosphate exit. (A) Exemplary trajectory of γ-phosphate exiting through the ATP binding site in Prp2. **(B)** Exemplary trajectory of γ-phosphate through exit channel between motif I and III of Prp43. **(C)** Example of γ-phosphate leaving complex through the ATP binding site in Prp43. **(D)** Motif I and III need to undergo only minor movements in order to enable exit of γ-phosphate.

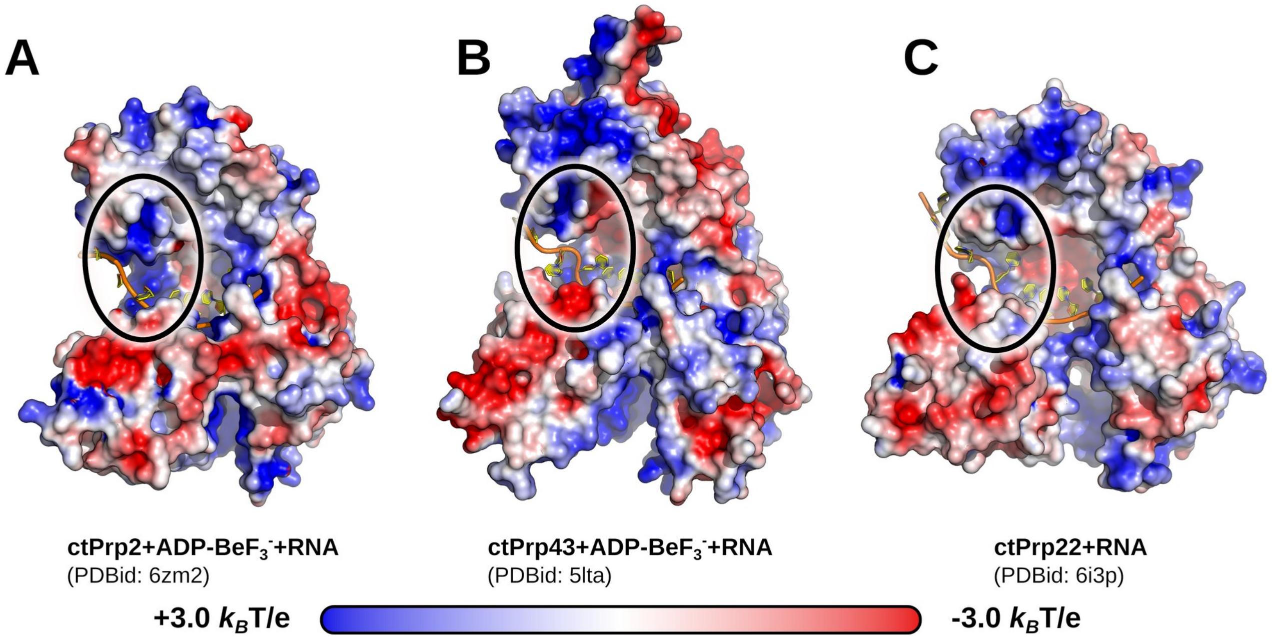

S8: Electrostatic potential of RNA-bound Prp2, Prp43 and Prp22 structures. Prp2 **(A)**, Prp43 **(B)** and Prp22 **(C)** are depicted as surface representation and colored based on their electrostatic potential. The RNA is displayed as a cartoon model. The region of interest close to the 5' RNA region kink is highlighted with a circle. The helix-bundle domain was omitted for clarity reasons. Prp2 exhibits the strongest positive charge of this region and likely influences the conformation of the 5' region.

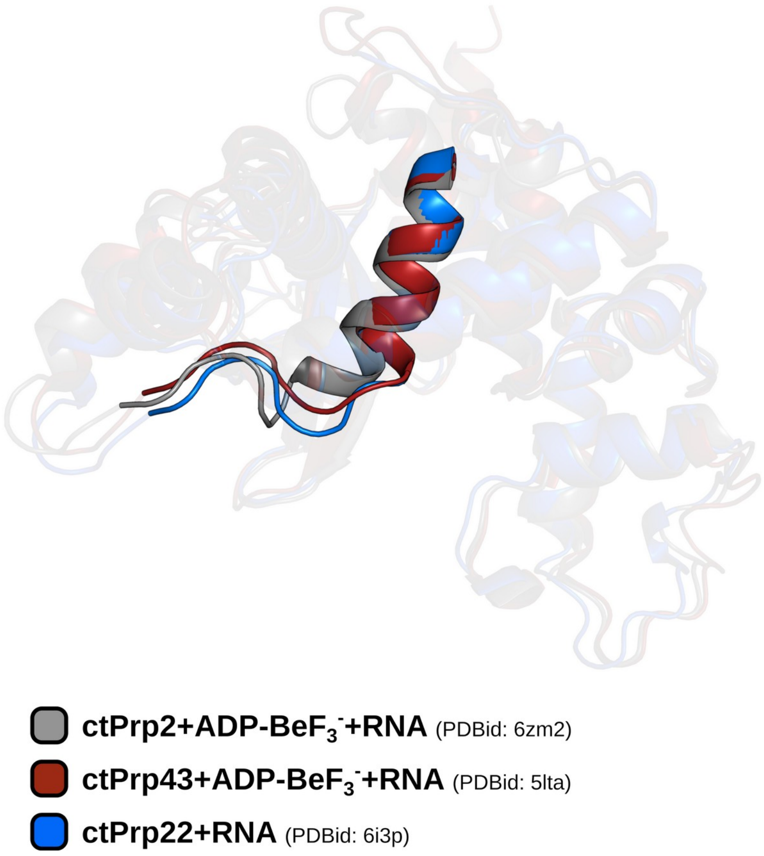

S9: Comparison of the C-terminal loop of Prp2, Prp43 and Prp22. Due to the insertion in the Prp2 C-terminal loop, the helix proceeding this loop is significantly longer and the loop itself has a unique conformation.

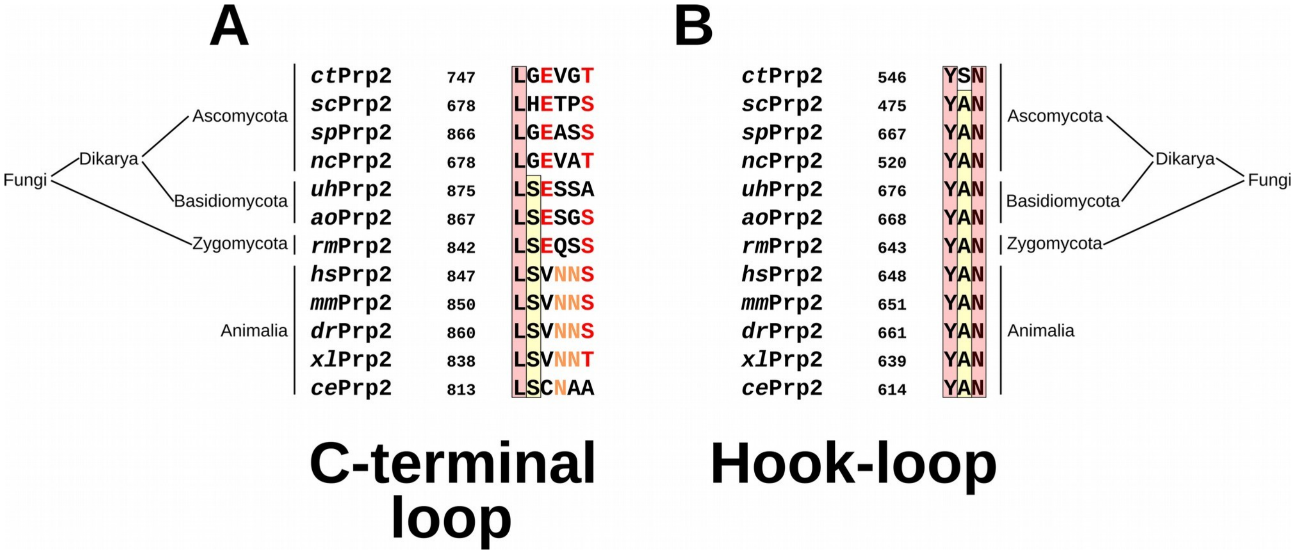

S10: Sequence conservation of Prp2 C-terminal loop and hook-loop in different organisms. Sequence alignment of the Prp2 C-terminal loop **(A)** and hook-loop **(B)** in *Chaetomium thermophilum*, *Saccaromyces cerevisiae*, *Saccaromyces pombe*, *Neurospora crassa*, *Ustilago hordei*, *Armillaria ostoyae*, *Rhizopus microsporus*, *Homo sapiens*, *Mus musculus*, *Danio rerio*, *Xenopus laevis* and *Caenorhabditis elegans*.

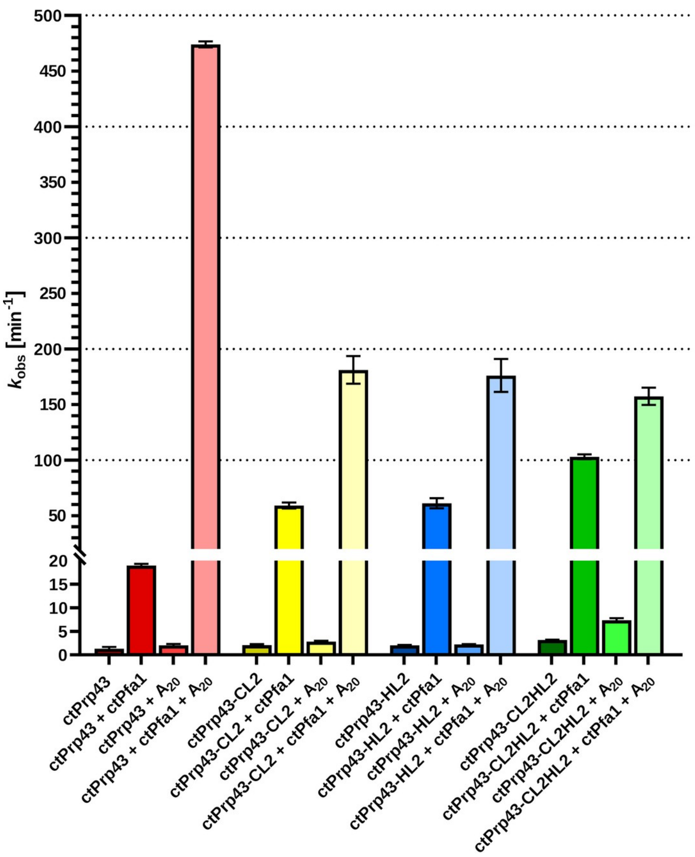

	k_{obs} [min^{-1}]	+/-
**ctPrp43**	1.32	0.34
**ctPrp43 + ctPfa1**	18.97	0.36
**ctPrp43 + A_{20}-RNA**	2.01	0.29
**ctPrp43 + ctPfa1 + A_{20}-RNA**	474.00	2.68
**ctPrp43-CL2**	2.06	0.22
**ctPrp43-CL2 + ctPfa1**	59.18	2.72
**ctPrp43-CL2 + A_{20}-RNA**	2.81	0.15
**ctPrp43-CL2 + ctPfa1 + A_{20}-RNA**	181.11	12.47
**ctPrp43-HL2**	2.04	0.07
**ctPrp43-HL2 + ctPfa1**	61.24	4.49
**ctPrp43-HL2 + A_{20}-RNA**	2.21	0.05
**ctPrp43-HL2 + ctPfa1 + A_{20}-RNA**	176.13	14.85
**ctPrp43-CL2HL2**	3.17	0.05
**ctPrp43-CL2HL2 + ctPfa1**	103.05	2.14
**ctPrp43-CL2HL2 + A_{20}-RNA**	7.38	0.42
**ctPrp43-CL2HL2 + ctPfa1 + A_{20}-RNA**	165.81	7.81

S11: ATPase activity measurements of ctPrp43 mutants. All measurements were performed with 2 mM ATP. In addition to the basal ATPase activity, all ctPrp43 constructs were analyzed as well in the presence of either 5-fold molar excess of the G-Patch motif of ctPfa1 or 10-fold molar excess of A_{20}-ssRNA or both. k_{obs} values are depicted in the right panel table. All measurements were performed in triplicates and the standard deviation is highlighted as error bars in the bar plot or stated as +/- in the table.

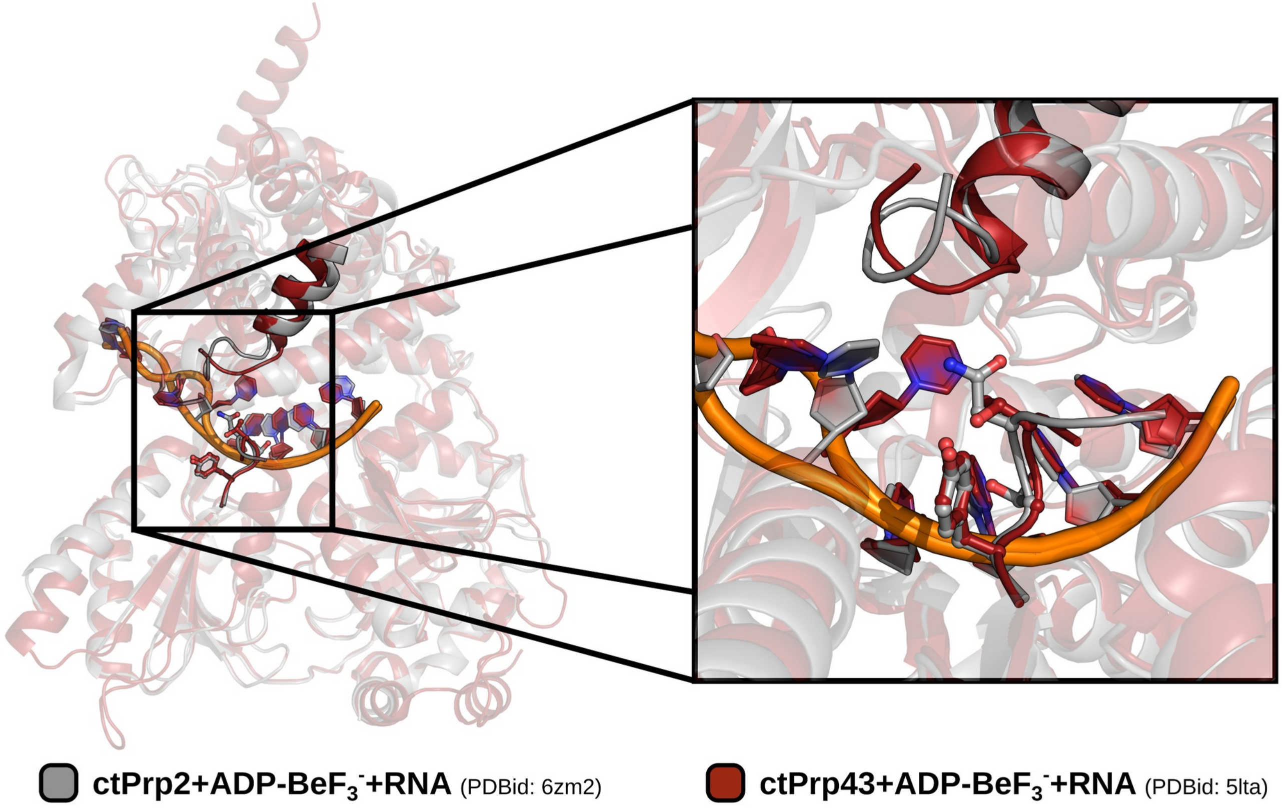

S12: Overview of hook-loop residues from ctPrp43 and ctPrp2 with respect to the C-terminal loop and bound RNA. Superposition of ctPrp2 and ctPrp43 via the RecA2 domain. C-terminal loops, hook-loops and ssRNA are highlighted.

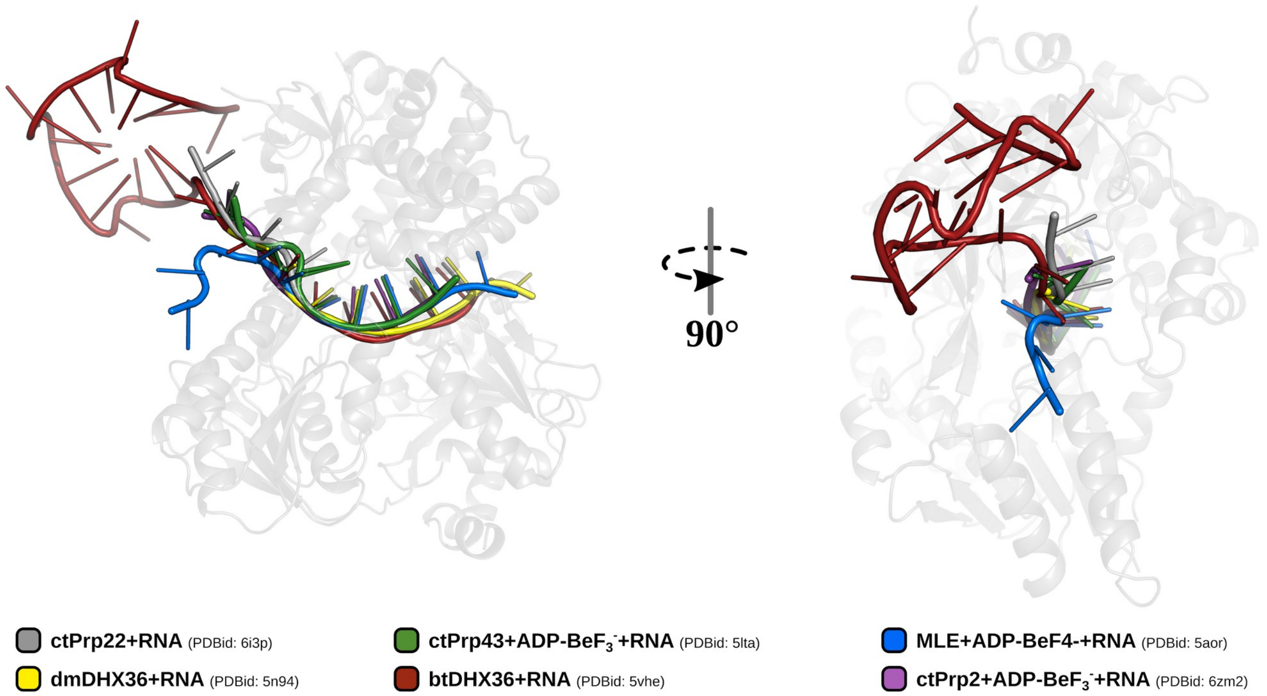

S13: Overview of different RNA-containing DExH-box ATPase complexes. All complexes were superimposed via the RecA2 domain and only ctPrp2 is displayed as a semi-transparent cartoon model. The 3' stacked region of the ssRNA shows a highly similar conformation in the different complexes, but the 5' region strongly differs.
